# Temporal dynamics of the fecal microbiome in wintering seagulls: a One Health perspective

**DOI:** 10.1186/s12864-026-12629-7

**Published:** 2026-02-12

**Authors:** Mingmei Wang, Yuanyuan Qu, Xiaofang Ma, Yeshun Fan, Chi Zhang, Jing Li, Xiaoxuan Liu, Zhen Wang, Jing Li, Yingdi Wang, Tianlong Zhang, Dianfeng Chu, Jie Liu, Yisong Li

**Affiliations:** 1 School of Public Health, Qingdao University, Qingdao, China; 2 Qingdao Municipal Center for Disease Control and Prevention, Qingdao, China; 3 The Affiliated Hospital of Qingdao University, Qingdao, China; 4 Department of Laboratory Medicine, Qingdao Municipal Hospital, University of Health and Rehabilitation Sciences, Qingdao, China; 5 Yebio BioEngineering Co. Ltd of Qingdao, Qingdao, China

**Keywords:** Gut microbiome, Temporal dynamics, Pathogenic bacteria, Wintering seagull, One Health

## Abstract

**Background:**

Migratory birds serve as critical reservoirs for zoonotic pathogens, with transmission risks significantly amplified by anthropogenic activities such as recreational feeding. Despite their role in disseminating pathogens, the temporal dynamics of the gut microbiota in wintering seagulls under sustained human contact remain poorly characterized within a One Health framework.

**Results:**

In this study, we conducted semi-monthly fecal sampling of black-headed gulls overwintering at a coastal tourism hotspot during consecutive wintering periods. Full-length 16S rRNA PacBio HiFi sequencing revealed remarkable microbial diversity and skewed distributions within the fecal communities. Although stochastic processes dominated microbial community assembly, temporal dynamics were observed as significant fluctuations in diversity indices, shifts in taxa prevalence, and episodic blooms of specific bacteria, reflecting signatures of foraging activities and anthropogenic interventions, particularly the provision of supplemental human food. Notably, 53 putative species-level pathogens were identified, with 11 of these exhibiting potential for cross-host transmission between migratory gulls and local inhabitants.

**Conclusions:**

Overall, this study provides a comprehensive One Health perspective on the gut microbiome of wintering migratory seagulls, offering valuable reference information for future wildlife management and public health protection.

**Supplementary Information:**

The online version contains supplementary material available at 10.1186/s12864-026-12629-7.

## Background

Pathogen transmission is a core challenge for One Health implementation [1, 2]. Zoonoses originating from wildlife, particularly bacterial agents, represent an increasing and significant threat to global health and economies [3, 4]. Although direct transmission of avian-borne pathogens to humans remains infrequently documented, migratory birds are still recognized as critical reservoirs for pathogens such as avian influenza, *Salmonella*, and antimicrobial-resistant *Escherichia coli* [5–7], and as asymptomatic carriers that could maintain and disseminate these agents across long distances, diverse ecosystems, and multiple host populations [8, 9]. The emergence of such zoonotic threats is associated with a range of underlying causal factors. These include the dense aggregations of animals at dwindling stopover sites, which create ecological hotspots for intensified pathogen transmission within wildlife populations, and the increasing human proximity into these habitats, which significantly amplifies the risk of cross-species spillover from wildlife reservoirs to humans [2, 3].

Seagulls, which seasonally overwinter in East Asia and North Africa from Siberia, are among the most prevalent migratory bird species in both inland and coastal regions of China. Surveillance data have confirmed their role in harboring putative bacterial pathogens, thereby establishing seagulls as reservoirs for a wide range of pathogenic agents [10–12]. Under natural conditions, direct contact between humans and wild birds is generally limited, and human exposure to wildlife-associated pathogens occurs primarily through indirect environmental pathways, including water, air, soil, and vegetation [8, 13, 14].

Recently, however, intentional seagull feeding has emerged as a prominent recreational activity in multiple urban centers. Such human-mediated food provisioning can locally elevate seagull population densities and substantially increase the frequency of close human-seagull interactions, thereby enhancing opportunities for pathogen exposure via both direct contact and intensified fecal contamination of public spaces and urban water bodies [11, 15]. Although direct evidence linking seagull feeding to disease outbreaks remains limited, the combination of higher host density and intensified human-wildlife contact suggests a plausible increase in transmission risk. Consequently, systematic pathogen surveillance in seagull populations can contribute to robust risk assessment and early warning efforts relevant to public health. Moreover, ​the widespread supplementary feeding​ by humans may also disrupt wild bird gut microbiota dynamics [16–18]. Elucidating temporal dynamics in seagull gut microbiota under anthropogenic influence would also advance our understanding of wildlife health and conservation strategies.

The present study aimed to investigate the temporal dynamics within the gut microbiome of wintering seagulls under ​sustained anthropogenic contact, and to evaluate localized public health risks through a One Health framework [1]. For this purpose, we collected fecal samples ​semi-monthly​ over ​two consecutive wintering periods​ from black-headed gulls overwintering at Qingdao Bay. This coastal site supports substantial gull aggregations during migration cycles and is ​a popular tourism destination due to gull-feeding activities. Leveraging full-length 16S rRNA PacBio HiFi sequencing, we ​achieved the first species-level profiling​ of dynamic bacterial diversity in wintering gulls, ​and evaluated​ the transmission potential ​among​ these gulls, the environment, and local residents.

## Methods

### Ethics statement

The whole project was approved by the Ethics Committee of Medical College of Qingdao University (QDU-AEC-2024388 and QDU-HEC-2024246), and was performed in accordance with the relevant guidelines and regulations.

### Sample collection and processing

**Fig. 1:**
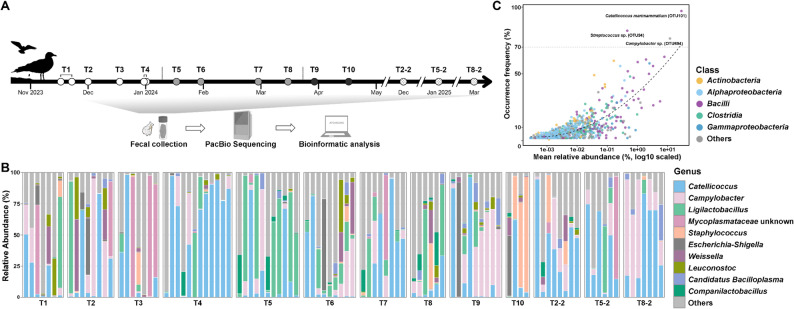
Study design and community structure in seagull feces. **A **Temporal sampling design across two consecutive overwintering periods. Circles denote distinct sampling timepoints. White, gray, and black circles represent the early-, mid-, and late-stages of the initial overwintering period (2023–2024), respectively, while dotted circles represent sampling timepoints during the subsequent overwintering period (2024–2025).​ **B **Relative abundance of the ten most abundant genera. **C **Scatter plot showing the relationship between mean relative abundance and occurrence frequency of species-level OTUs. The X-axis indicates mean relative abundance (%) and Y-axis represents the occurrence (% of samples) throughout the sampling timepoints. Points represent individual OTUs, colored by different taxonomic classes. Dashed curve indicates fitted distribution trend

This study was conducted at Zhanqiao Beach (120.32°E, 36.06°N) in Qingdao, China, where seagull fecal samples were systematically collected from November 2023 to April 2025 (Fig. 1A). Sampling spanned two consecutive wintering periods: the initial series (T1-T10) represented semi-monthly collections during 2023–2024, while three additional interannual validation groups (T2-2, T5-2, and T8-2) were sampled at the timepoints corresponding to T2, T5, and T8, respectively, during the subsequent wintering period (2024–2025).

Fresh seagull fecal samples were aseptically collected using sterile swabs and immediately transferred to pre-chilled sterile containers. Concurrent meteorological parameters, including ambient temperature and weather conditions, were recorded at each sampling event. To quantify anthropogenic activity, specifically public engagement in seagull feeding, we tracked daily Baidu search volumes [19] for the Chinese phrase “feeding seagulls” in Qingdao during wintering periods.​.

To establish a reference dataset for potential pathogen sharing, fecal samples from local residents were collected from the physical examination cohort at Qingdao Municipal Hospital using sterile containers, while sputum specimens were obtained from patients in the Emergency Department (ED) of the Affiliated Hospital of Qingdao University. These samples serve as a baseline for identifying overlapping pathogens and potential threats, not as evidence for direct transmission pathways. Seawater samples (5 L) were collected from the intertidal zone of the beach during wintering period, and vacuum-filtered using a 0.22 μm polytetrafluoroethylene (PTFE) membrane to concentrate the microbiomes.

All samples were immediately refrigerated at 4 ℃ and transported to the laboratory within two hours, then stored at -80 ℃ until testing.

### DNA extraction and full-length 16S rRNA gene sequencing

All samples were extracted using the TIANamp Stool DNA Kit (TIANGEN, Beijing, China), according to the manufacturer’s instructions. The mitochondrial cytochrome oxidase subunit I (COI) gene was amplified with the primers specifically designed for birds [20] with modifications to identify seagull species (F: 5’-CACGAATAAACAACATRAGCTTYTG-3’; R: 5’-CTGGGTGDCCRAARAATC-3’). To avoid inter-host variability in the fecal microbiome, only samples confirmed to be from *Chroicocephalus ridibundus* (black-headed gull) were subjected to full-length (V1-V9 regions) 16S rRNA gene sequencing. The primers used were 27 F (5′- AGAGTTTGATCCTGGCTCAG-3′) and 1492R (5′-GNTACCTTGTTACGACTT-3′). Sequencing was performed by Novogene Company (Beijing, China) using the PacBio Revio platform. The HiFi reads were generated using SMRT Link software and demultiplexed using lima. The 5,632,802 HiFi reads (average length = 1,467 bp) obtained were analyzed using the DADA2 package, according to the workflow previously described [21] with slight modifications, including quality control, denoising, and amplicon sequence variants (ASVs) identification. Three samples containing fewer than 30,000 reads were excluded from downstream analyses. To ensure analytical robustness and to minimize the impact of potential sequencing noise, we implemented a multi-step filtering process. This included the removal of non-bacterial sequences (chloroplast, mitochondrial, eukaryotic, archaeal), singletons (ASVs appearing in only one sample), and low-abundance ASVs (represented by fewer than 25 total reads) prior to downstream analyses. Thereafter, ASVs were clustered into species-level OTUs using VSEARCH at a 98.65% identity threshold [22, 23]. This consolidation was implemented to avoid artificial taxon inflation arising from intragenomic 16S rRNA gene heterogeneity and to reduce stochastic noise, thereby enhancing the ecological signal of the temporal dynamics. Taxonomic annotation of these OTUs was performed against the Silva v138 database [24]. For higher-resolution identification, representative sequences were further queried against the NCBI 16S rRNA database via BLASTn [25], using an E-value cutoff of 1e-5 and a minimum alignment length of 1,200 bp to ensure reliable species-level annotation. Human-derived specimens (fecal and sputum samples) and seawater were processed in parallel using identical bioinformatic parameters and quality thresholds for methodological consistency.

### Putative pathogen detection

To identify the potential bacterial pathogens, the representative sequence of each species-level OTU was firstly compared with the pathogen database in multiple bacterial pathogen detection (MBPD) [26] using BLASTn with a 98.65% similarity threshold and e-value < 1 × 10^−5^. ​To ensure maximum reliability, the resulting taxonomic assignment was then compared with the bacterial pathogen lists reported by KARIUS^®^ [27], the Risk Group Database from the American Biological Safety Association [28], and a customized database constructed by Chen et al. [29]. An OTU was categorized as a putative pathogen only if present in at least one of these three databases and was further classified as zoonotic or animal-specific according to MBPD annotations. Importantly, since 16S rRNA gene sequencing cannot distinguish between pathogenic and non-pathogenic at the strain level, nor determine viability or infectivity, these identified taxa represent potential pathogens based on taxonomic affiliation rather than confirmed disease risk. In addition, only OTUs initially defined within the seagull-specific dataset were considered potential shared OTUs when evaluating pathogen distribution among humans, seawater, and seagulls. This approach was adopted to maintain taxonomic consistency and to avoid potential artifacts introduced by cross-dataset re-clustering [30].

### Downstream bioinformatic and statistical analysis

Most analyses were conducted using R software (version 4.4.3). For all statistical analyses, a *P*-valu*e* < 0.05 was considered statistically significant. α-diversity indices (Shannon and Chao1 richness) were calculated with the vegan package based on filtered and normalized counts after rarefaction to the minimum sequencing depth. Beta diversity patterns were assessed via principal coordinates analysis (PCoA) on the basis of the Bray-Curtis dissimilarity. Distance-based redundancy analysis (dbRDA) was performed with the vegan package [31]. PERMANOVA was performed using the adonis function of the vegan package to evaluate the significance of sampling timepoints with 999 permutations based on Bray-Curtis dissimilarity. Additionally, the homogeneity of multivariate dispersion was assessed using the betadisper function.

The community assembly processes were quantified based on the null model [32, 33]. The β-nearest taxon index (βNTI) and Raup-Crick index (RC) were calculated with 1,000 randomizations to determine the relative dominance of the factors in shaping the composition of the seagull fecal bacterial community (homogeneous selection, βNTI < -2; heterogeneous selection, βNTI > 2; homogeneous dispersal, RC < -0.95 and |βNTI| < 2; dispersal limitation, RC > 0.95 and |βNTI| < 2; and stochastic drift, |RC| < 0.95 and |βNTI| < 2). For each timepoint, the relative contribution of each process was calculated as the percentage of pairwise community comparisons assigned to that process.

Species-level OTU prevalence dynamics were modeled using Generalized Additive Models (GAMs) with a binomial error distribution and logit link function [34]. The smoothing basis was set at k = 6 to extract primary trends while suppressing high-frequency stochastic noise. A taxon was considered to exhibit significant temporal fluctuations only if the model achieved statistical significance (*P* < 0.05) and the predicted prevalence shift met a minimum amplitude threshold of 0.3. To identify typical dynamic patterns, we applied stringent structural criteria where global monotonic patterns (increasing/decreasing) were defined by a consistent directional shift without any opposing fluctuations. For complex profiles such as hump-shaped, U-shaped, and oscillating patterns, every individual temporal segment of the fluctuation was also required to meet the 0.3 threshold. Dynamics failing to fulfill these amplitude or structural requirements were excluded from further analysis.

To identify significant temporal correlations and dynamic features, we performed extended local similarity analysis (eLSA) [35] based on the relative abundance of each dominant OTUs using the following parameters: -s 10 -r 12 -d 0 -p theo, with the default significance threshold (*P* < 0.05) for theoretical *p*-value estimation. Specifically, outliers in the OTU relative abundance data were identified at each timepoint using the interquartile range (IQR) method. Values exceeding 1.5 times the IQR from the first or third quartiles, along with any other missing values, were imputed with the group mean. Local similarity values with |LS| ≥ 0.6 were selected for further analysis. Time series were clustered using z-score normalized seasonal profiles calculated from group means with the repr_seas_profile function from the TSrepr package (v1.1.0) [36]. The optimal number of clusters was determined by minimizing the Davies-Bouldin index [37]. Clustering of microbial communities was conducted using the Markov Clustering Algorithm (MCL) in the Cytoscape plugin clusterMaker [38], using only positive local similarity (LS) values as input.

## Results

### Study design and characteristics of the sequencing data

To elucidate the temporal dynamics of seagull feces, we executed a systematic sampling campaign spanning two consecutive wintering periods at Zhanqiao Beach in Qingdao. During the initial wintering period (November 2023 to April 2024), semi-monthly sampling was implemented, yielding a comprehensive dataset comprising ten sequential timepoints (Fig. 1A). ​The sampling period could be divided into early (T1-T4), middle (T5-T8), and late (T9-T10) stages of wintering, broadly reflecting the local tourism conditions: pre-tourism, peak-tourism, and post-tourism, respectively (Figure S1).​ The average temperature during this period was 7.04 ± 6.28 ℃, exhibiting an initial decrease, followed by a stabilization period, and a subsequent increase. In the subsequent wintering period (2024–2025), targeted sampling was conducted at three corresponding timepoints to capture interannual variability.

A total of 168 fecal samples were collected. COI sequencing revealed that the local seagull population was predominantly composed of *Chroicocephalus ridibundus* (also known as black-headed gull; 88.69%), while other identified species included *Larus crassirostris* (3.57%), *Larus glaucoide*s (2.38%), *Larus smithsonianu*s (2.38%), *Larus fuscus* (1.19%), and other species at lower frequencies. By analyzing 102 fecal samples collected from black-headed gulls, after quality filtering, a total of 4,355,372 high-quality full-length 16S rRNA gene reads were generated with an average of 42,700 reads per sample (Table S1), resulting in 2,654 ASVs. The rarefaction curves of ASV counts plateaued across all samples, suggesting that sequencing depth was sufficient to capture the majority of ASV-level diversity (Figure S2). After clustering ASVs at 98.65% identity, 1,126 species-level OTUs were retained for downstream analysis (Table S2).

### Fecal bacterial community composition in wintering seagulls

Overall, a total of 17 phyla, 32 classes, 425 genera, and 1,126 species-level OTUs were detected in black-headed gull feces, of which 718 were putative new species. Among these, *Firmicutes* emerged as the dominant phylum with an average relative abundance of 73.34%, followed by *Campylobacterota* (14.17%) and *Proteobacteria* (9.77%) (Figure S3). In addition, three dominant genera collectively occupied > 50% relative abundance, including *Catellicoccus* (29.05%; primarily represented by *C*. *marimammalium*, 28.86%), *Campylobacter* (12.70%; mainly an unidentifiable species, OTU694, 12.01%), and *Ligilactobacillus* (11.25%; predominantly *L. aviarius*, 7.86%) (Fig. 1B).

Notably, regarding the prevalence, the majority of the species-level OTUs exhibited limited distribution (Fig. 1C). Merely eleven (representing 0.98% of the total OTUs) demonstrated occurrence frequencies > 50%, of which seven belonged to class *Bacilli*. Among these, only three OTUs (0.27% of the total) exhibited broad distributions (prevalence > 70%). The most prevalent was *C. marimammalium* (OTU101, 97.1%), followed by an unclassified *Streptococcus* (OTU94, 82.4%) and an unclassified *Campylobacter* (OTU694, 76.5%). Conversely, low-prevalence taxa dominated the community, with 82.68% of the total OTUs occurring at frequencies below 10%, suggesting remarkable diversity and limited community stability for the vast majority of taxa.

### Temporal variations in microbial diversity and community assembly processes

**Fig. 2:**
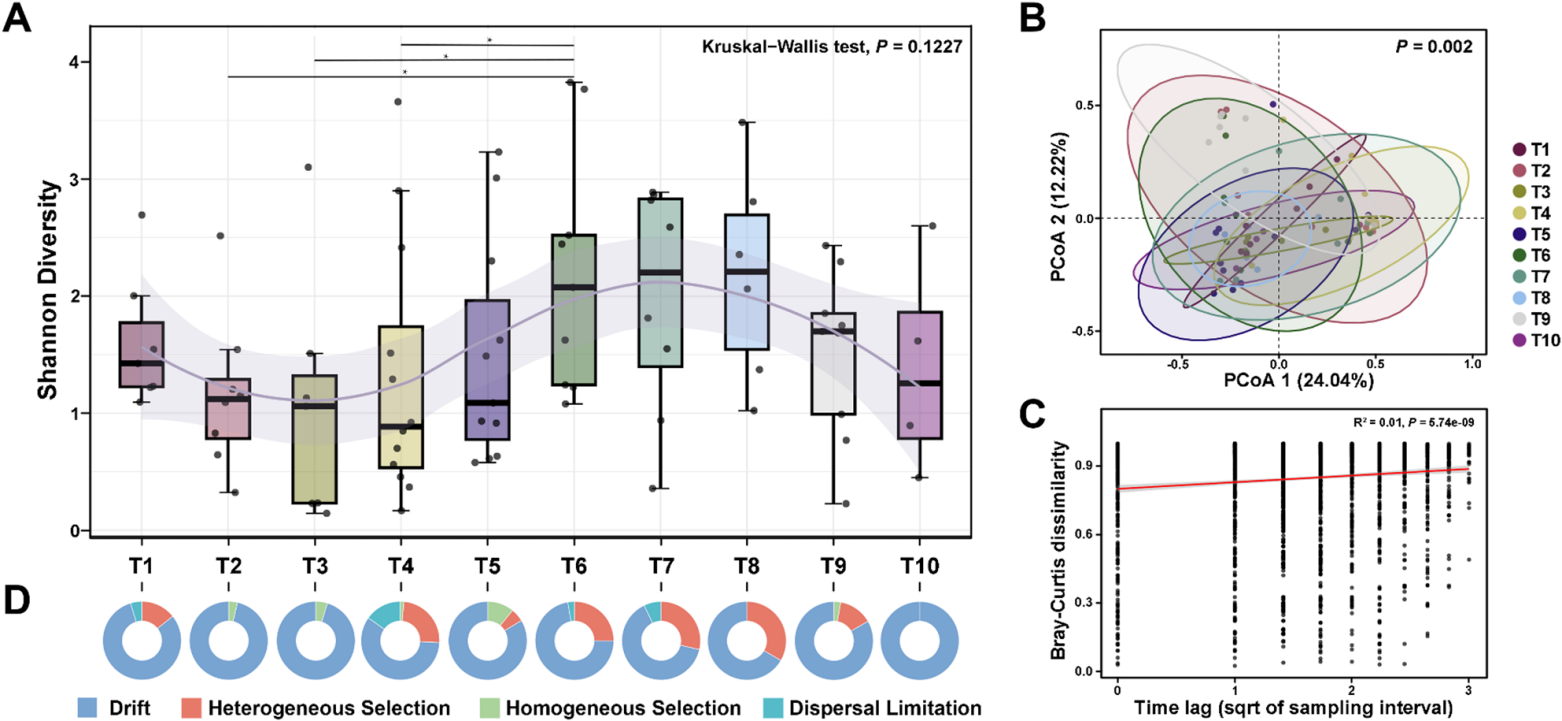
Temporal variations in microbial diversity during the overwintering period. **A **α‑diversity (Shannon) over time. Statistical significance was tested by Kruskal-Wallis test, followed by multiple comparisons using Wilcoxon rank-sum test (*, *P* < 0.05). The ​light purple trend line represents loess smooth regression estimates, with shaded area representing 95% confidence interval. **B **The PCoA plot based on Bray-Curtis dissimilarity showing the compositional differences among different sampling timepoints. *P*-values were obtained from PERMANOVA. **C **Linear regression between community dissimilarity and collection date interval.​​ Each point denotes pairwise dissimilarity between samples separated by a specific time lag. The Pearson’s correlation coefficient of the linear regression is presented. **D **The relative contribution of different ecological processes to the assembly of the bacterial community in seagull feces

Although the Kruskal-Wallis test revealed no statistically significant differences across all sampling timepoints (*P* = 0.1227), the temporal dynamics of Shannon diversity showed a non-monotonic trajectory, characterized by an initial decrease, followed by a gradual increase, and ultimately a decline (Fig. 2A). The median values of lowest and highest Shannon indices were significantly distinct, occurring at T4 and T8, respectively. Variation in Chao1 richness exhibited a similar pattern (Figure S4).​ To further investigate temporal heterogeneity at the community level, beta-diversity was analyzed. PERMANOVA revealed significant shifts in community composition across consecutive sampling intervals (*P* = 0.002; Fig. 2B), while multivariate dispersion remained homogeneous (*P* = 0.743). Distance-based redundancy analysis (dbRDA) revealed that temporal variables explained 19.7% of beta-diversity variation (*P* = 0.001), while weather conditions​ contributed to an additional 5.26% (*P* = 0.016). Time-lag regression analysis revealed a significant but weak positive relationship between temporal distance and Bray-Curtis dissimilarity (R^2^ = 0.01, *P* < 0.001; Fig. 2C).

Furthermore, null-model analysis revealed that the assembly of the seagull fecal bacterial community was primarily governed by stochastic processes. Throughout the wintering period (T1-T10), ecological drift was identified as the predominant assembly process, with an average contribution of 80.19% (Fig. 2D and Figure S5; Table S3). Despite this overall stochastic dominance, the relative influence of deterministic selection exhibited temporal fluctuations. The estimated contribution of selection reached an initial peak at T4 (25.76%), followed by a decrease at T5. Subsequently, its influence rose during the mid-wintering stage, reaching a maximum of 33.33% at T8 before declining thereafter. These results suggest that while the seagull fecal microbiota is characterized by a strong stochastic signature, it also undergoes subtle temporal shifts in the relative balance of assembly processes across different wintering stages.

### Prevalence dynamics of fecal bacteria

**Fig. 3:**
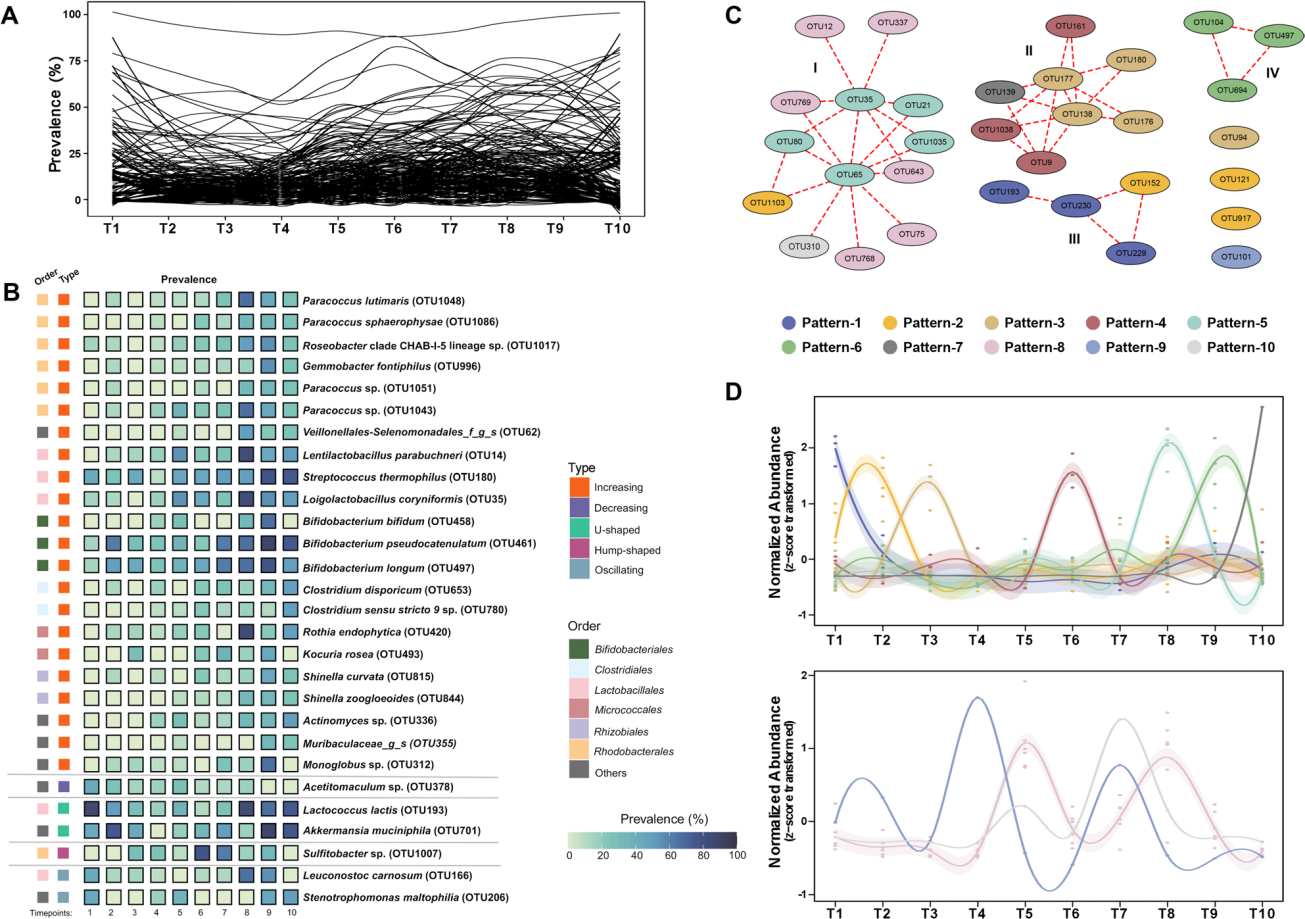
Temporal dynamics of fecal microbiome in overwintering seagulls. **A **Line graph depicting the heterogeneous and dynamic prevalence of 1,126 species-level OTUs across ten sampling timepoints. Each black line represents one OTU. **B **Prevalence dynamics of species-level OTUs clustered into five temporal types. **C **and** D **Association network and time series representations of fecal microbes. Each node represents a species-level OTU and each edge represents a positive correlation between paired OTUs. I/II/III/IV represent different association modules. The trend line depicts the loess-smoothed, z-score-normalized relative abundance of each pattern over time. Details of the species-level OTUs were listed in Table S4

Throughout the entire wintering period, the prevalence of fecal bacteria varied substantially (Fig. 3A), with only 14 species-level OTUs consistently detected across all sampling timepoints. Further longitudinal analysis of the entire microbial community identified five distinct prevalence patterns among all OTUs (Fig. 3B and Figure S6). Twenty-two OTUs across ten bacterial orders exhibited a significant increase in prevalence over time. This upward trend was predominantly driven by members of the *Rhodobacterales* (6 OTUs belonging to *Paracoccus*, *Gemmobacter* and *Roseobacter*), *Bifidobacteriales* (3 OTUs: *Bifidobacterium bifidum*, *B. pseudocatenulatum*, and *B. longum*), and *Lactobacillales* (3 OTUs: *Lentilactobacillus parabuchneri*, *Loigolactobacillus coryniformis*, and *Streptococcus thermophilus*), which collectively accounted for over half (12/22) of the increasing taxa. The remaining increasing taxa were distributed across orders such as *Micrococcales* (2 OTUs: *Kocuria rosea* and *Rothia endophytica*), *Clostridiales* (2 OTUs: *Clostridium sensu stricto* 9 OTU780 and *C. disporicum*), *Rhizobiales* (2 OTUs: *Shinella curvata* and *S. zoogloeoide*), and et al.

In contrast, the other four patterns were represented by a small number of OTUs, including one decreasing (*Acetitomaculum* OTU378), two oscillating (*Leuconostoc carnosum* and *Stenotrophomonas maltophilia*) with multiple fluctuations, one hump-shaped (*Sulfitobacter* OTU1007) peaking during the mid-wintering stage, and two U-shaped (*Lactococcus lactis* and *Akkermansia muciniphila*).

### Temporal abundance dynamics of dominant species-level OTUs

We next analyzed dominant species-level OTUs (defined as > 0.1% relative abundance in > 30% of samples per timepoint, present in ≥ 4 timepoints) to assess whether they underwent correlated shifts within the community. A total of ten dynamic patterns were identified among 32 OTUs, categorized into ​two broad trend types, and clustered into ​four modules driven by positive temporal microbe-microbe correlations (Figs. 3C and D; Table S4).​​ Three-quarters of the species exhibited transient blooms, characterized by unimodal peaks at specific timepoints against otherwise stable baseline abundances. For example, the OTUs that first exploded predominantly belonged to *Enterobacteriaceae* (2 of 3 OTUs), including common enteric pathogens *Escherichia fergusonii* (OTU229) and *Shigella dysenteriae* (OTU230); all *Streptococcaceae* OTUs displayed early-stage explosions, with *Lactococcus lactis* (OTU193) appearing first, followed sequentially by *Lactococcus chungangensis* (OTU121), and finally *Streptococcus macedonicus* (OTU176), *Streptococcus lutetiensis* (OTU177), *Streptococcus thermophilu*s (OTU180), and one unclassified *Streptococcus* OTU94; mid-stage explosions were dominated by *Lactobacillaceae* (OTU9, OTU21, OTU35, OTU65, OTU161; 5 of 8 OTUs), while late-stage explosions showed greater phylogenetic diversity, spanning four distinct families.

The remaining eight species were characterized by oscillatory dynamics. Of these, six OTUs, including those from *Clostridium saudiense* (OTU643), *Ligilactobacillus aviarius* (OTU75), *Paucilactobacillus nenjiangensis* (OTU12), *Intestinibacter bartlettii* (OTU768), *Romboutsia timonensis* (OTU769), and an unclassified species from family *Eubacteriaceae* (OTU337), exhibited ​a mid-stage abundance plateau with a shallow U-shaped trajectory. *Catellicoccus marimammalium* (OTU101) exhibited substantial fluctuations featuring initial ascent, mid-stage oscillations, and eventual depletion, while OTU310 from *Eubacteriaceae* displayed mid-stage fluctuations with an upward trend, followed by a gradual decline.

### Potential pathogenic bacteria in seagull feces

**Fig. 4:**
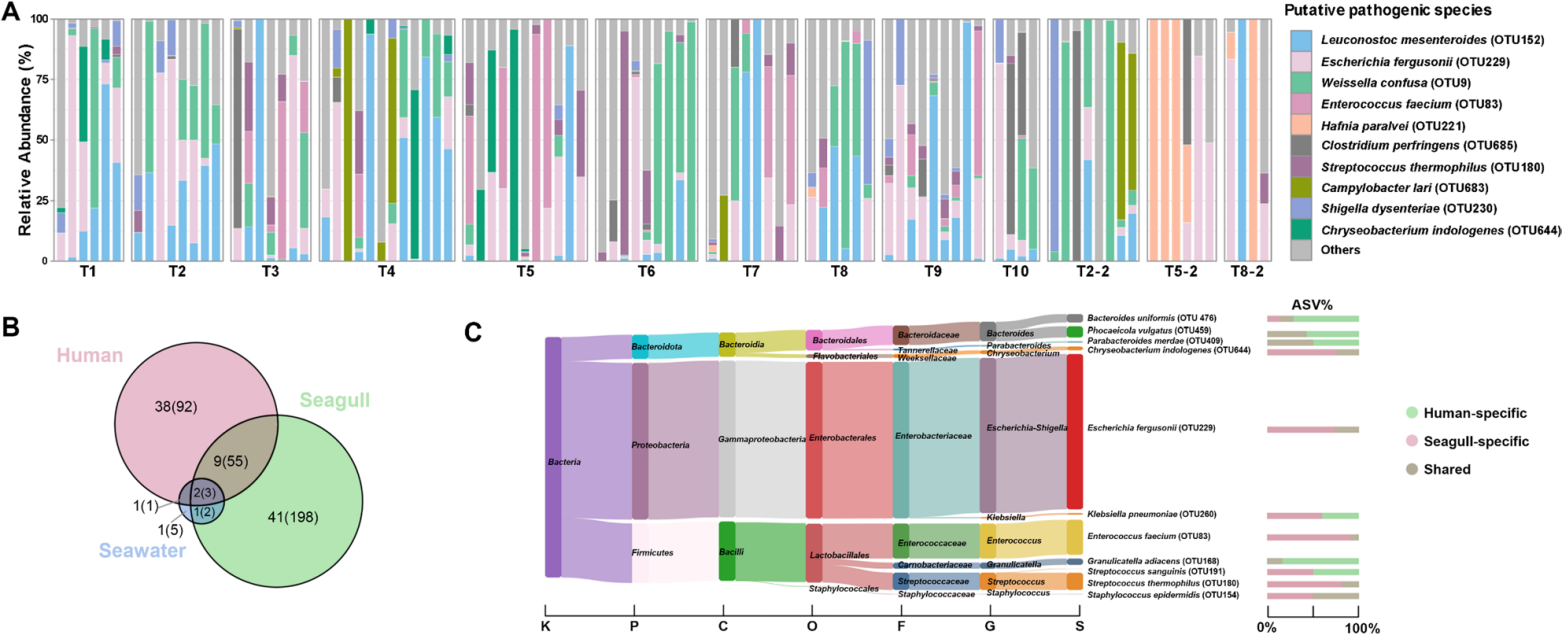
Community structure and distribution of putative pathogenic species-level OTUs. **A **Relative abundance of the ten most abundant putative pathogenic OTUs. **B **Venn plot showing overlap of putative pathogenic OTUs among seagulls, local residents, and seawater. Values in parentheses represent ASV counts. **C **Sankey diagram illustrating the taxonomic distribution of putative pathogenic OTUs shared between humans and seagulls, with integrated horizontal stacked bar chart quantifying ASV origin per species. The X-axis at the bottom left represents taxonomic levels

Across all seagull fecal samples, 53 (4.71%) putative pathogenic (or opportunistic) species-level OTUs spanning six phyla were identified, including 20 zoonotic and 33 animal-associated agents (Table S5). Each sample contained 0 to 17 such OTUs (mean: 5; median: 5). Specifically, while these potentially pathogenic OTUs exhibited a low relative abundance (< 2%, averaging 0.54%) in the majority of samples (61.76%), six samples showed levels exceeding 50%, with a maximum of 95.98%. Notably, 79.25% (42/53) and 71.70% (38/53) of these taxa ranked among the top 50% in overall abundance and prevalence, respectively. Moreover, the top ten putative pathogenic OTUs, comprising four zoonotic and six animal-associated agents, collectively accounted for 76.01% of total abundance of these taxa (Fig. 4A). Among potentially zoonotic OTUs, *Escherichia fergusonii* was dominant, with an average relative abundance of 16.13% among putative pathogens and 2.52% of the whole microbial community, followed by *Clostridium perfringens* (4.30%/0.13%), *Streptococcus thermophilus* (3.78%/0.46%), and *Shigella dysenteriae* (3.33%/0.50%). The top animal-associated taxa included *Leuconostoc mesenteroides* (16.92%/1.02%), *Weissella confusa* (13.96%/2.42%), *Enterococcus faecium* (6.35%/0.76%), *Hafnia paralvei* (4.60%/0.52%), *Campylobacter lari* (3.45%/0.06%), and *Chryseobacterium indologenes* (3.18%/0.08%). Specifically, the α-diversity of these potentially pathogenic (or opportunistic) OTUs also exhibited a decline in early-stage, but then exhibited a fluctuating upward trend (Figure S7).

To assess seagull-associated health risks, putative pathogenic species-level OTUs shared among seagulls, local residents, and seawater were reanalyzed using a Venn diagram (Fig. 4B). A total of 11 OTUs were identified that were shared between humans and seagulls​, accounting for 22% (11/50) of human-associated pathogens and 20.75% (11/53; Table S5) of seagull-associated pathogens. ASV-level analysis showed 38.41% (58/151) and 22.48% (58/258) sharing, respectively. Notably​, nine of these OTUs comprised identical ASVs shared between both hosts, suggesting conserved genetic signatures at the 16S rRNA level and highlighting the putative microbial connectivity at human-seagull interfaces (Fig. 4C). Although these identical sequences do not definitively prove transmission, they highlight the potential for microbial exchange and the presence of common exposure sources. Among these, *Escherichia fergusonii* predominated in abundance, followed by *Enterococcus faecium* and *Streptococcus thermophilus*. *Phocaeicola vulgatus* and *Parabacteroides merdae* were also detected from seawater, suggesting the widespread presence of these taxa. In addition, although seawater contained fewer putative pathogens (five in total), 80% were traceable to human/seagull sources.

### Interannual variation of the seagull fecal microbiome

Overall, the α-diversity of the seagull fecal microbiome showed no significant change across consecutive wintering periods (Figure S8). While PERMANOVA revealed significant shifts in β-diversity between years (*P* = 0.013; Figure S9), the effect size was very small (R^2^ = 0.02), indicating that interannual variation accounts for only 2% of the total community variance. This suggests that while year-to-year differences are statistically detectable, the overall structure of the seagull gut microbiota remained relatively stable across years. In addition, these minor interannual differences appeared to be period-specific. For instance, no detectable variations were observed at T2 and T5, whereas significant divergences in both α- and β-diversity emerged at T8 (*P* < 0.05; Figure S10). This late-stage divergence may be attributed to the cumulative effects of urban environmental stressors and anthropogenic disturbances. However, a total of 19 novel species-level OTUs and 64 novel ASVs were detected, suggesting potentially different ecological adaptations or functional innovations.

## Discussion

Seagulls greatly benefit from human activities and often occur in popular tourist destinations [39, 40], and simultaneously, they constitute important reservoirs for pathogens that threaten aquatic ecosystems and public health [8, 12, 41]. While their complex gut bacterial communities likely sustain their ecologies and facilitate their biological functions, distribution, and diversity, these communities are strongly influenced by environmental factors and host diet [42]. At overwintering sites, tourist activities such as feeding and observation largely intensify human-gull interactions. This intimate interface facilitates bidirectional microbial transmission, posing risks to human populations while simultaneously altering or perturbing gull intestinal microbiota. As the dominant gull species at Zhanqiao Beach in winter, the black-headed gull demonstrates a high degree of adaptability to external environments, exhibiting a gut microbiome distinct from that of other local birds [43, 44]. Our study therefore characterizes the temporal dynamics of black-headed gull microbiota, identifies possible anthropogenic perturbations, and traces potential dissemination pathways across seagull-human-environment interfaces, thereby highlighting associated risks within a One Health perspective.

### Highly skewed distributions and stochastic drift drive wintering seagull microbiome diversity

By integrating full-length 16S rRNA sequencing with longitudinal sampling, our study achieved enhanced taxonomic resolution of gut microbiota in migratory seagulls, uncovering previously unresolved temporal dynamics during wintering.​ Through large-scale PacBio sequencing of 102 samples, we identified 1,126 microbial species-level OTUs and 2,654 ASVs, including 718 putatively novel species requiring further investigation. Notably, given the stringent data filtering criteria applied in our pipeline to ensure sequence reliability, the actual diversity of novel species may still be underestimated. This effort established a comprehensive profile of the gut microbial diversity in black-headed gulls throughout a complete wintering cycle.

The bacterial community exhibited pronounced prevalence skewness. While only three species-level OTUs were broadly distributed, over 80% of taxa were transient specialists (detection frequency < 10%) that collectively accounted for a substantial portion of the phylogenetic diversity. *Catellicoccus marimammalium* dominated as a core gut symbiont in black-headed gulls, displaying both the highest relative abundance (28.86%) and universal prevalence (> 95%) across all sampled individuals. These findings align with prior studies on black-headed gulls from other geographic regions, which suggest its potential role in enhancing environmental adaptability and suppressing viral replication [44–46]. Following *C. marimammalium*, the next most prevalent OTUs belonged to the genera *Streptococcus* and *Campylobacter*, both of which harbor ​well-known or opportunistic pathogens ​associated with​ various diseases [10, 47, 48].

The overwhelming predominance of low-prevalence taxa, coupled with episodic species-specific blooms, suggests a community structure potentially characterized by stochastic assembly with high susceptibility to extrinsic drivers, such as nutrient pulses from anthropogenic feeding or other environmental factors. This is consistent with our null-model analysis, which estimated that ecological drift contributed approximately 80% to the community composition. However, this high proportion of stochasticity should be interpreted with caution, as null-model frameworks are highly sensitive to data preprocessing, where rarefaction, the filtering of rare taxa, and overall community similarity can artificially inflate the perceived influence of stochastic drift [49, 50]. ​Nevertheless, this high gut microbiota plasticity could provide migratory seagulls with adaptive advantages, potentially enhancing their ability to utilize diverse local prey or conferring resistance against novel pathogens encountered during migration and wintering [51].

### Seagull microbiota dynamics: foraging and anthropogenic interventions

Our results demonstrate, for the first time, significant temporal fluctuations in α-diversity​ throughout the wintering period. Although tourism intensity was not identified as a statistically significant driver in dbRDA (*P* > 0.05), the pronounced elevation in diversity and strengthened heterogeneous selection observed during the peak-tourism mid-stage (T5-T8) may nonetheless reflect the ecological consequences of intensified human-seagull interactions. This process is likely mediated by cumulative anthropogenic feeding and other density-dependent factors, consistent with the well-established role of diet as the primary determinant of the avian gut microbiota [52–54].

Consistent microbial shifts, probably driven by the interplay of natural foraging and anthropogenic effects, were observed throughout the wintering period. Notably, the progressive increase in the prevalence of *Roseobacteraceae* likely reflects the accumulation of marine-associated taxa through opportunistic predation on algae, zooplankton, and fish [55, 56].​ Furthermore, the prevalence of *Bifidobacteriales* and *Lactobacillaceae* also exhibited significant upward trajectories. This dynamic is likely a metabolic adaptation to starch-enriched diets provisioned by tourists (e.g., bread fragments), as dietary starch supplementation has been shown to selectively promote the proliferation of these fermentative taxa in both mammalian and avian gut microbiomes [57–59]. ​Given their sustained prevalence and established metabolic relevance to the shifting dietary profile, these taxa appear to represent stable, diet-responsive residents rather than mere transient environmental acquisitions.

However, our eLSA network analysis revealed that this deterministic selection may also occur alongside highly dynamic community abundances, as three-quarters of the dominant OTUs exhibited transient blooms characterized by unimodal peaks at specific timepoints. These transient groups, along with those displaying oscillatory dynamics, likely reflect short-term responses to episodic environmental fluctuations or dietary pulses. It should be mentioned that due to the highly skewed distributions of the OTUs, we have removed outliers and imputed missing values with group means prior to eLSA, which may have artificially inflated temporal correlations and introduced potential bias. Nevertheless, these results suggest that while the seagull microbiota is characterized by high diversity and transient successions, anthropogenic interventions could also provide selective pressures to facilitate the gradual establishment of a set of functional, diet-responsive residents within the seagull gut.

Additionally, although overall α-diversity showed no significant differences between arriving and departing seagulls, ​distinct structural reorganization occurred in microbial communities. Interannual comparisons revealed minimal early/mid-wintering divergence but pronounced late-stage variation, perhaps suggesting that cumulative environmental and anthropogenic drivers, such as sustained climatic fluctuations or tourist provisioning, combined with other stochastic disturbances may restructure the bacterial communities in the seagull gut. Nevertheless, the overwhelming dominance of ecological drift (~ 80%), though potentially overestimated, likely constrains both the magnitude and persistence of such deterministic influences, as stochastic forces could rapidly drive community reassembly toward randomized structures.

### Health risks arising from seagull-human interactions: a One Health perspective

Although there was little direct clinical evidence in previous studies, birds, particularly free-living migratory species, are recognized as critical pathogen reservoirs and sentinels due to their enhanced long-distance dispersal capacity and ecological plasticity. These traits significantly amplify global disease spread, making migratory birds a critical public health concern [8, 10]. Of these, migratory seagulls that forage on anthropogenic food sources and waste in tourist areas act as biological intermediaries, effectively linking the microbial communities of humans, poultry, and livestock. This spatiotemporal overlap facilitates potential cross-species transmission, a process that necessitates integrated surveillance within One Health frameworks. While previous studies primarily utilized culture-based methods and focused on targeted pathogens, recent advances in molecular diagnostics, particularly next-generation sequencing (NGS), now enable comprehensive profiling of gut pathogenic bacterial spectra [12, 41, 44, 48, 60].

Our high-resolution PacBio full-length 16S rRNA sequencing revealed potential public health risks, highlighted by the presence of putative shared pathogens (common OTUs and ASVs) between seagulls and local residents. The detection of shared taxa, including clinically relevant species such as *Escherichia fergusonii* and *Enterococcus faecium*, suggests a potential for microbial exchange at the human-seagull interface. Notably, *E. fergusonii* is recognized for its zoonotic pathogenicity and has been documented in food-chain transmission [61, 62], particularly in poultry [63, 64]. It is also known to harbor antibiotic resistance genes, which can complicate clinical treatment [65]. In the present study, we also found that *E. fergusonii* underwent an abundance surge, further suggesting its potential risk. In addition, *E. faecium*, a core member of the intestinal microbiota of humans and animals, functions as an opportunistic pathogen capable of causing life-threatening infections, particularly among hospitalized patients [66, 67]. The third most abundant shared pathogen is *S. thermophilus*. Despite its longstanding ‘Generally Recognized As Safe’ status and extensive use in yogurt and cheese production, close phylogenetic ties to pathogenic streptococci raise concerns about its virulence potential [68]. However, there are currently no definitive reports of its pathogenicity in seagulls or humans. Critically, the detection of putative human-associated opportunists (*Parabacteroides merdae* and *Phocaeicola vulgatus*) in seawater suggested anthropogenic fecal contamination and subsequent ingestion by seagulls [69–71]. This evidence supports the existence of a ​human-environment-seagull fecal-oral transmission cycle, highlighting the imperative for stringent sewage and waste management to mitigate potential escalating public health risks. Other potential clinically significant pathogens detected in seagull feces include *Klebsiella pneumoniae*, *Staphylococcus epidermidis*, *Granulicatella adiacens*, etc., all of which are recognized as putative agents of critical human infections. However, it should be noted that the shared ASVs observed across hosts and environments in this study may reflect a shared occurrence resulting from common exposure rather than confirmed transmission. Future studies incorporating whole-genome sequencing (WGS) or strain-level cultivation are necessary to provide definitive epidemiological evidence of pathogen spillover.

Furthermore, unlike the overall bacterial community diversity which peaked at the mid-wintering stage and then declined, the α-diversity of putative pathogens exhibited an increasing trend from mid-stage onward, potentially reflecting cumulative exposure effects resulting from complex anthropogenic contacts. Collectively, these findings seem to highlight a heightened potential health risk associated with migratory seagulls, particularly during periods of direct human-seagull interaction. Given the significant temporal and individual variations observed, long-term and large-scale epidemiological surveillance within One Health frameworks remains essential.​ However, it is important to note that the relatively low prevalence and abundance of these putative pathogens suggest that while these findings warrant further scientific attention, they should be interpreted with caution and do not currently necessitate widespread public health alarm.

### Limitations

Our study has some limitations. First, confinement to a single beach may inadequately represent the ecological heterogeneity of seagull microbiomes across diverse habitats. Future work should incorporate multi-regional sampling to enhance ecological representativeness. Second, absence of minimally disturbed control groups (e.g., natural habitats with negligible anthropogenic exposure) limits definitive attribution of human activity impacts. Third, although we employed an internet-derived search index to quantify human-seagull interaction intensity alongside meteorological parameters (e.g., temperature, weather), we did not measure other critical confounders like gull diet composition and natural prey availability. These factors could independently alter the microbiota and potentially covary with human activity, thus to some extent confounding our interpretation of the specific role of human provisioning. Further investigation is required to isolate the definitive impact of human feeding from background ecological variation dynamics. Finally, although PacBio full-length 16S rRNA sequencing offers high resolution, it has inherent limitations for definitive species delineation and may not always strictly correspond to genomic species boundaries. Furthermore, though our stringent filtering of singletons and low-abundance ASVs may ensure analytical robustness by minimizing sequencing artifacts, it may underestimate the rare biosphere and its role in stochastic assembly. Future metagenomic sequencing, particularly through the recovery of Metagenome-Assembled Genomes (MAGs), would be essential to provide genomic validation for putative novel taxa and to better elucidate their functional dynamics. This approach will also enable a strain-level identification of virulence factors and infectivity, thereby confirming whether these putative pathogens pose a true disease risk. Such genomic insights, combined with the characterization of antimicrobial resistance genes (ARGs), will provide a more definitive framework for addressing global public health threats posed by wild bird-associated microbiomes.

## Conclusions

In conclusion, this work provides, to our knowledge, the first in-depth species-level analysis of temporal dynamics in the fecal microbiome of wintering seagulls. Our study reveals a remarkably diverse and stochastically assembled microbial community in black-headed gulls, characterized by temporal fluctuations of specific taxa and episodic species-specific blooms. These dynamics are likely influenced by foraging behaviors and anthropogenic interventions. Critically, our findings highlight the necessity of continued monitoring of potential bidirectional pathogen transmission during frequent human-seagull interactions. By resolving the gut microbiome landscapes of migratory seagulls, this study advances the One Health perspective and provides a valuable reference for future wildlife management and public health protection.

## Supplementary Information

Supplementary Material 1.

Supplementary Material 2.

## Data Availability

Raw reads are available on the Sequence Read Archive of the National Center of Biotechnology Information (NCBI) under the BioProject number PRJNA1313872.

## References

[1] Cunningham AA, Daszak P, Wood JLN. One Health, emerging infectious diseases and wildlife: two decades of progress? Philos Trans R Soc Lond B Biol Sci. 2017;372(1725). 10.1098/rstb.2016.0167. 10.1098/rstb.2016.0167 PMC5468692 28584175

[2] Daszak P, Cunningham AA, Hyatt AD. Emerging infectious diseases of wildlife-threats to biodiversity and human health. Science. 2000;287(5452):443–9. 10.1126/science.287.5452.443. 10642539 10.1126/science.287.5452.443

[3] Altizer S, Bartel R, Han BA. Animal migration and infectious disease risk. Science. 2011;331(6015):296–302. 10.1126/science.1194694. 21252339 10.1126/science.1194694

[4] Jones KE, Patel NG, Levy MA, Storeygard A, Balk D, Gittleman JL, Daszak P. Global trends in emerging infectious diseases. Nature. 2008;451(7181):990–3. 10.1038/nature06536. 18288193 10.1038/nature06536 PMC5960580

[5] Lin Y, Dong X, Sun R, Wu J, Tian L, Rao D, Zhang L, Yang K. Migratory birds-one major source of environmental antibiotic resistance around Qinghai Lake, China. Sci Total Environ. 2020;739:139758. 10.1016/j.scitotenv.2020.139758. 32540654 10.1016/j.scitotenv.2020.139758 PMC7260505

[6] Olsen B, Munster VJ, Wallensten A, Waldenström J, Osterhaus AD, Fouchier RA. Global patterns of influenza a virus in wild birds. Science. 2006;312(5772):384–8. 10.1126/science.1122438. 16627734 10.1126/science.1122438

[7] Wigley P. *Salmonella* and salmonellosis in wild birds. Anim (Basel). 2024;14(23). 10.3390/ani14233533. 10.3390/ani14233533 PMC11640726 39682498

[8] Tsiodras S, Kelesidis T, Kelesidis I, Bauchinger U, Falagas ME. Human infections associated with wild birds. J Infect. 2008;56(2):83–98. 10.1016/j.jinf.2007.11.001. 18096237 10.1016/j.jinf.2007.11.001 PMC7172416

[9] Viana DS, Santamaría L, Figuerola J. Migratory birds as global dispersal vectors. Trends Ecol Evol. 2016;31(10):763–75. 10.1016/j.tree.2016.07.005. 27507683 10.1016/j.tree.2016.07.005

[10] Kobuszewska A, Wysok B. Pathogenic bacteria in free-living birds, and its public health significance. Anim (Basel). 2024;14(6). 10.3390/ani14060968. 10.3390/ani14060968 PMC10967383 38540066

[11] Wu B, Wang XC, Dzakpasu M. Genetic characterization of fecal impacts of seagull migration on an urban scenery lake. Water Res. 2017;117:27–36. 10.1016/j.watres.2017.03.041. 28364653 10.1016/j.watres.2017.03.041

[12] Liao F, Qian J, Yang R, Gu W, Li R, Yang T, Fu X, Yuan B, Zhang Y. Metagenomics of gut microbiome for migratory seagulls in Kunming city revealed the potential public risk to human health. BMC Genomics. 2023;24(1):269. 10.1186/s12864-023-09379-1. 37208617 10.1186/s12864-023-09379-1 PMC10196292

[13] Ishii S, Nakamura T, Ozawa S, Kobayashi A, Sano D, Okabe S. Water quality monitoring and risk assessment by simultaneous multipathogen quantification. Environ Sci Technol. 2014;48(9):4744–9. 10.1021/es500578s. 24702133 10.1021/es500578s

[14] Kobayashi M, Zhang Q, Segawa T, Maeda M, Hirano R, Okabe S, Ishii S. Temporal dynamics of *Campylobacter* and *Arcobacter* in a freshwater lake that receives fecal inputs from migratory geese. Water Res. 2022;217:118397. 10.1016/j.watres.2022.118397. 35421690 10.1016/j.watres.2022.118397

[15] Araújo S, Henriques IS, Leandro SM, Alves A, Pereira A, Correia A. Gulls identified as major source of fecal pollution in coastal waters: a microbial source tracking study. Sci Total Environ. 2014;470–471:84–91. 10.1016/j.scitotenv.2013.09.075. 24140684 10.1016/j.scitotenv.2013.09.075

[16] Davidson GL, Wiley N, Cooke AC, Johnson CN, Fouhy F, Reichert MS, de la Hera I, Crane JMS, Kulahci IG, Ross RP, et al. Diet induces parallel changes to the gut microbiota and problem solving performance in a wild bird. Sci Rep. 2020;10(1):20783. 10.1038/s41598-020-77256-y. 33247162 10.1038/s41598-020-77256-y PMC7699645

[17] Teyssier A, Matthysen E, Hudin NS, de Neve L, White J, Lens L. Diet contributes to urban-induced alterations in gut microbiota: experimental evidence from a wild passerine. Proc Biol Sci. 2020;287(1920):20192182. 10.1098/rspb.2019.2182. 10.1098/rspb.2019.2182 PMC7031670 32019440

[18] Jones I, Marsh K, Handby TM, Hopkins K, Slezacek J, Bearhop S, Harrison XA. The influence of diet on gut microbiome and body mass dynamics in a capital-breeding migratory bird. PeerJ. 2023;11:e16682. 10.7717/peerj.16682. 38130921 10.7717/peerj.16682 PMC10734429

[19] Baidu Inc. Baidu Index platform. Baidu. https://index.baidu.com/v2/index.html. (accessed July 30, 2025).

[20] de Melo AA, Nunes R, Telles MPC. Same information, new applications: revisiting primers for the avian COI gene and improving DNA barcoding identification. Org Divers Evol. 2021;21(3):599–614. 10.1007/s13127-021-00507-x.

[21] Callahan BJ, Wong J, Heiner C, Oh S, Theriot CM, Gulati AS, McGill SK, Dougherty MK. High-throughput amplicon sequencing of the full-length 16S rRNA gene with single-nucleotide resolution. Nucleic Acids Res. 2019;47(18):e103. 10.1093/nar/gkz569. 31269198 10.1093/nar/gkz569 PMC6765137

[22] Kim M, Oh HS, Park SC, Chun J. Towards a taxonomic coherence between average nucleotide identity and 16S rRNA gene sequence similarity for species demarcation of prokaryotes. Int J Syst Evol Microbiol. 2014;64(Pt 2):346–51. 10.1099/ijs.0.059774-0. 24505072 10.1099/ijs.0.059774-0

[23] Rognes T, Flouri T, Nichols B, Quince C, Mahé F. VSEARCH: a versatile open source tool for metagenomics. PeerJ. 2016;4:e2584. 10.7717/peerj.2584. 27781170 10.7717/peerj.2584 PMC5075697

[24] Quast C, Pruesse E, Yilmaz P, Gerken J, Schweer T, Yarza P, Peplies J, Glöckner FO. The SILVA ribosomal RNA gene database project: improved data processing and web-based tools. Nucleic Acids Res. 2013;41(Database issue):D590–596. 10.1093/nar/gks1219. 23193283 10.1093/nar/gks1219 PMC3531112

[25] Camacho C, Coulouris G, Avagyan V, Ma N, Papadopoulos J, Bealer K, Madden TL. BLAST+: architecture and applications. BMC Bioinformatics. 2009;10:421. 10.1186/1471-2105-10-421. 20003500 10.1186/1471-2105-10-421 PMC2803857

[26] Yang X, Jiang G, Zhang Y, Wang N, Zhang Y, Wang X, et al. MBPD: A multiple bacterial pathogen detection pipeline for One Health practices. iMeta. 2023;2(1):e82. 10.1002/imt2.82. 38868336 10.1002/imt2.82 PMC10989770

[27] Karius Inc. Pathogen List. Karius. https://kariusdx.com/our-solution/pathogens. (accessed July 30, 2025).

[28] American Biological Safety Association (ABSA). Risk Groups Database. ABSA. https://my.absa.org/Riskgroups. (accessed July 30, 2025).

[29] Chen Q, An X, Li H, Su J, Ma Y, Zhu YG. Long-term field application of sewage sludge increases the abundance of antibiotic resistance genes in soil. Environ Int. 2016;92–93:1–10. 10.1016/j.envint.2016.03.026. 27043971 10.1016/j.envint.2016.03.026

[30] He Y, Caporaso JG, Jiang XT, Sheng HF, Huse SM, Rideout JR, Edgar RC, Kopylova E, Walters WA, Knight R, Zhou HW. Stability of operational taxonomic units: an important but neglected property for analyzing microbial diversity. Microbiome. 2015;3:20. 10.1186/s40168-015-0081-x. 25995836 10.1186/s40168-015-0081-x PMC4438525

[31] Legendre P, Anderson MJ. Distance-based redundancy analysis: testing multispecies responses in multifactorial ecological experiments. Ecol Monogr. 1999;69(1):1–24. 10.2307/2657192.

[32] Stegen JC, Lin X, Fredrickson JK, Chen X, Kennedy DW, Murray CJ, Rockhold ML, Konopka A. Quantifying community assembly processes and identifying features that impose them. ISME J. 2013;7(11):2069–79. 10.1038/ismej.2013.93. 23739053 10.1038/ismej.2013.93 PMC3806266

[33] Stegen JC, Lin X, Fredrickson JK, Konopka AE. Estimating and mapping ecological processes influencing microbial community assembly. Front Microbiol. 2015;6:370. 10.3389/fmicb.2015.00370. 25983725 10.3389/fmicb.2015.00370 PMC4416444

[34] Wood SN. Generalized additive models: an introduction with R. New York: Chapman and Hall/CRC; 2017. 10.1201/9781315370279.

[35] Xia LC, Steele JA, Cram JA, Cardon ZG, Simmons SL, Vallino JJ, Fuhrman JA, Sun F. Extended local similarity analysis (eLSA) of microbial community and other time series data with replicates. BMC Syst Biol. 2011;5(Suppl 2):S15. 10.1186/1752-0509-5-s2-s15. 22784572 10.1186/1752-0509-5-S2-S15 PMC3287481

[36] Laurinec P. TSrepr R package: time series representations. J Open Source Softw. 2018;3(23):577. 10.21105/joss.00577.

[37] Davies DL, Bouldin DW. A cluster separation measure. IEEE Trans Pattern Anal Mach Intell. 1979;1(2):224–7. 10.1109/TPAMI.1979.4766909. 21868852

[38] Morris JH, Apeltsin L, Newman AM, Baumbach J, Wittkop T, Su G, Bader GD, Ferrin TE. ClusterMaker: a multi-algorithm clustering plugin for cytoscape. BMC Bioinformatics. 2011;12:436. 10.1186/1471-2105-12-436. 22070249 10.1186/1471-2105-12-436 PMC3262844

[39] Feng C, Liang W. Behavioral responses of black-headed gulls (*Chroicocephalus ridibundus)* to artificial provisioning in China. Glob Ecol Conserv. 2020;21:e00873. 10.1016/j.gecco.2019.e00873.

[40] Xu H, Zhao X, Jia R, Chen L, Yang Z, Zhang, G. Behavioral plasticity mediates adaptation to changes in food provisioning following the COVID-19 lockdown in black-headed gulls (Larus ridibundus). Front Ecol Evol. 2022;10:1013244. 10.3389/fevo.2022.1013244.

[41] Lu J, Santo Domingo JW, Lamendella R, Edge T, Hill S. Phylogenetic diversity and molecular detection of bacteria in gull feces. Appl Environ Microbiol. 2008;74(13):3969–76. 10.1128/aem.00019-08. 18469128 10.1128/AEM.00019-08 PMC2446513

[42] Bodawatta KH, Hird SM, Grond K, Poulsen M, Jønsson KA. Avian gut microbiomes taking flight. Trends Microbiol. 2022;30(3):268–80. 10.1016/j.tim.2021.07.003. 34393028 10.1016/j.tim.2021.07.003

[43] Bonnedahl J, Drobni P, Johansson A, Hernandez J, Melhus A, Stedt J, Olsen B, Drobni M. Characterization, and comparison, of human clinical and black-headed gull *(Larus ridibundus*) extended-spectrum beta-lactamase-producing bacterial isolates from Kalmar, on the southeast coast of Sweden. J Antimicrob Chemother. 2010;65(9):1939–44. 10.1093/jac/dkq222. 20615928 10.1093/jac/dkq222

[44] Luo Q, Gao H, Xiang Y, Li J, Dong L, Wang X, Liu F, Guo Y, Shen C, Ding Q, et al. The dynamics of microbiome and virome in migratory birds of southwest China. NPJ Biofilms Microbiomes. 2025;11(1):64. 10.1038/s41522-025-00703-z. 40268958 10.1038/s41522-025-00703-z PMC12018928

[45] Indykiewicz P, Andrzejewska M, Minias P, Śpica D, Kowalski J. Prevalence and antibiotic resistance of *Campylobacter* spp. In urban and rural black-headed gulls *Chroicocephalus ridibundus*. EcoHealth. 2021;18(2):147–56. 10.1007/s10393-021-01540-0. 34478007 10.1007/s10393-021-01540-0 PMC8463336

[46] Ryu H, Griffith JF, Khan IU, Hill S, Edge TA, Toledo-Hernandez C, Gonzalez-Nieves J, Santo Domingo J. Comparison of gull feces-specific assays targeting the 16S rRNA genes of *Catellicoccus marimammalium* and *Streptococcus* spp. Appl Environ Microbiol. 2012;78(6):1909–16. 10.1128/aem.07192-11. 22226950 10.1128/AEM.07192-11 PMC3298170

[47] Smoglica C, Graziosi G, De Angelis D, Lupini C, Festino A, Catelli E, Vergara A, Di Francesco CE. Wild birds as drivers of *Salmonella* braenderup and multidrug resistant bacteria in wetlands of northern Italy. Transbound Emerg Dis. 2024;2024:6462849. 10.1155/2024/6462849. 10.1155/2024/6462849 PMC12016999 40303189

[48] Wu S, Jia R, Wang Y, Li J, Li Y, Wang L, Wang Y, Liu C, Jia EM, Wang Y, et al. Prevalence, diversity, and virulence of *Campylobacter* carried by migratory birds at four major habitats in China. Pathogens. 2024;13(3). 10.3390/pathogens13030230. 10.3390/pathogens13030230 PMC10975922 38535573

[49] Chase JM, Kraft NJ, Smith KG, Vellend M, Inouye BD. Using null models to disentangle variation in community dissimilarity from variation in α-diversity. Ecosphere. 2011;2(2):1–11. 10.1890/ES10-00117.1.

[50] Zhou J, Ning D. Stochastic community assembly: does it matter in microbial ecology? Microbiol Mol Biol Rev. 2017;81(4). 10.1128/mmbr.00002-17. 10.1128/MMBR.00002-17 PMC5706748 29021219

[51] Quinn JT, Hamilton DJ. Variation in diet of *Semipalmated sandpipers* (*Calidris pusilla*) during stopover in the upper Bay of Fundy, Canada. Can J Zool. 2012;90(9):1181–90. 10.1139/z2012-086.

[52] Grond K, Lanctot RB, Jumpponen A, Sandercock BK. Recruitment and establishment of the gut microbiome in arctic shorebirds. FEMS Microbiol Ecol. 2017;93(12). 10.1093/femsec/fix142. 10.1093/femsec/fix142 29069418

[53] Grond K, Santo Domingo JW, Lanctot RB, Jumpponen A, Bentzen RL, Boldenow ML, Brown SC, Casler B, Cunningham JA, Doll AC, et al. Composition and drivers of gut microbial communities in arctic-breeding shorebirds. Front Microbiol. 2019;10:2258. 10.3389/fmicb.2019.02258. 31649627 10.3389/fmicb.2019.02258 PMC6795060

[54] Sun F, Chen J, Liu K, Tang M, Yang Y. The avian gut microbiota: Diversity, influencing factors, and future directions. Front Microbiol. 2022;13:934272. 10.3389/fmicb.2022.934272. 35992664 10.3389/fmicb.2022.934272 PMC9389168

[55] Buchan A, González JM, Moran MA. Overview of the marine *Roseobacter* lineage. Appl Environ Microbiol. 2005;71(10):5665–77. 10.1128/aem.71.10.5665-5677.2005. 16204474 10.1128/AEM.71.10.5665-5677.2005 PMC1265941

[56] Wagner-Döbler I, Biebl H. Environmental biology of the marine *Roseobacter* lineage. Annu Rev Microbiol. 2006;60:255–80. 10.1146/annurev.micro.60.080805.142115. 16719716 10.1146/annurev.micro.60.080805.142115

[57] Jung DH, Park CS. Resistant starch utilization by *Bifidobacterium*, the beneficial human gut bacteria. Food Sci Biotechnol. 2023;32(4):441–52. 10.1007/s10068-023-01253-w. 36911330 10.1007/s10068-023-01253-w PMC9992497

[58] Raspa F, Chessa S, Bergero D, Sacchi P, Ferrocino I, Cocolin L, Corvaglia MR, Moretti R, Cavallini D, Valle E. Microbiota characterization throughout the digestive tract of horses fed a high-fiber vs. a high-starch diet. Front Vet Sci. 2024;11:1386135. 10.3389/fvets.2024.1386135. 38807937 10.3389/fvets.2024.1386135 PMC11130486

[59] Wang W, Zheng S, Sharshov K, Sun H, Yang F, Wang X, Li L, Xiao Z. Metagenomic profiling of gut microbial communities in both wild and artificially reared Bar-headed goose (*Anser indicus*). Microbiologyopen. 2017;6(2). 10.1002/mbo3.429. 10.1002/mbo3.429 PMC5387313 27998035

[60] Wang L, Jia R, Ma R, Li J, Wu S, Fan Y, Zhao D, Chu D, Wang Y, Zhang G, et al. High throughput screening for human disease associated-pathogens and antimicrobial resistance genes in migratory birds at ten habitat sites in China. BMC Microbiol. 2025;25(1):355. 10.1186/s12866-025-04059-4. 40481427 10.1186/s12866-025-04059-4 PMC12143040

[61] Gaastra W, Kusters JG, van Duijkeren E, Lipman LJ. *Escherichia fergusonii*. Vet Microbiol. 2014;172(1–2):7–12. 10.1016/j.vetmic.2014.04.016. 24861842 10.1016/j.vetmic.2014.04.016

[62] Rayamajhi N, Cha SB, Shin SW, Jung BY, Lim SK, Yoo HS. Plasmid typing and resistance profiling of *Escherichia Fergusonii* and other *Enterobacteriaceae* isolates from South Korean farm animals. Appl Environ Microbiol. 2011;77(9):3163–6. 10.1128/aem.02188-10. 21398479 10.1128/AEM.02188-10 PMC3126392

[63] Forgetta V, Rempel H, Malouin F, Vaillancourt R Jr., Topp E, Dewar K, Diarra MS. Pathogenic and multidrug-resistant *Escherichia fergusonii* from broiler chicken. Poult Sci. 2012;91(2):512–25. 10.3382/ps.2011-01738. 22252367 10.3382/ps.2011-01738

[64] Oh JY, Kang MS, An BK, Shin EG, Kim MJ, Kwon JH, Kwon YK. Isolation and epidemiological characterization of heat-labile enterotoxin-producing *Escherichia fergusonii* from healthy chickens. Vet Microbiol. 2012;160(1–2):170–5. 10.1016/j.vetmic.2012.05.020. 22771038 10.1016/j.vetmic.2012.05.020

[65] Tang B, Chang J, Chen Y, Lin J, Xiao X, Xia X, Lin J, Yang H, Zhao G. *Escherichia fergusonii*, an underrated repository for antimicrobial resistance in food animals. Microbiol Spectr. 2022;10(1):e0161721. 10.1128/spectrum.01617-21. 35138151 10.1128/spectrum.01617-21 PMC8826826

[66] Gouliouris T, Coll F, Ludden C, Blane B, Raven KE, Naydenova P, Crawley C, Török ME, Enoch DA, Brown NM, et al. Quantifying acquisition and transmission of *Enterococcus faecium* using genomic surveillance. Nat Microbiol. 2021;6(1):103–11. 10.1038/s41564-020-00806-7. 33106672 10.1038/s41564-020-00806-7 PMC7610418

[67] Wei Y, Palacios Araya D, Palmer KL. *Enterococcus faecium*: evolution, adaptation, pathogenesis and emerging therapeutics. Nat Rev Microbiol. 2024;22(11):705–21. 10.1038/s41579-024-01058-6. 38890478 10.1038/s41579-024-01058-6 PMC12147861

[68] Bolotin A, Quinquis B, Renault P, Sorokin A, Ehrlich SD, Kulakauskas S, Lapidus A, Goltsman E, Mazur M, Pusch GD, et al. Complete sequence and comparative genome analysis of the dairy bacterium *Streptococcus thermophilus*. Nat Biotechnol. 2004;22(12):1554–8. 10.1038/nbt1034. 15543133 10.1038/nbt1034 PMC7416660

[69] Cui Y, Zhang L, Wang X, Yi Y, Shan Y, Liu B, Zhou Y, Lü X. Roles of intestinal *Parabacteroides* in human health and diseases. FEMS Microbiol Lett. 2022;369(1). 10.1093/femsle/fnac072. 10.1093/femsle/fnac072 35945336

[70] Jamaluddin NF, Brovkina O, Nor Rashid N, Al-Maleki AR, Lim YA, Tan MP, et al. Gut microbiota profiles of peninsular Malaysian populations are associated with urbanization and lifestyle. Sci Rep. 2025;15(1):24066. 10.1038/s41598-025-07117-z. 40617918 10.1038/s41598-025-07117-z PMC12228821

[71] Mills RH, Dulai PS, Vázquez-Baeza Y, Sauceda C, Daniel N, Gerner RR, et al. Multi-omics analyses of the ulcerative colitis gut microbiome link *Bacteroides vulgatus* proteases with disease severity. Nat Microbiol. 2022;7(2):262–76. 10.1038/s41564-021-01050-3. 35087228 10.1038/s41564-021-01050-3 PMC8852248

