# Supplementary material for: Temporal dynamics of the fecal microbiome in wintering seagulls: a One Health perspective
Source: BMC Genomics. 2026 Feb 12;27:191. doi: 10.1186/s12864-026-12629-7 (PMC12903670; doi:10.1186/s12864-026-12629-7)
Supplement: Supplementary file 2 — Supplementary Material 2. [file 12864_2026_12629_MOESM2_ESM.pdf]

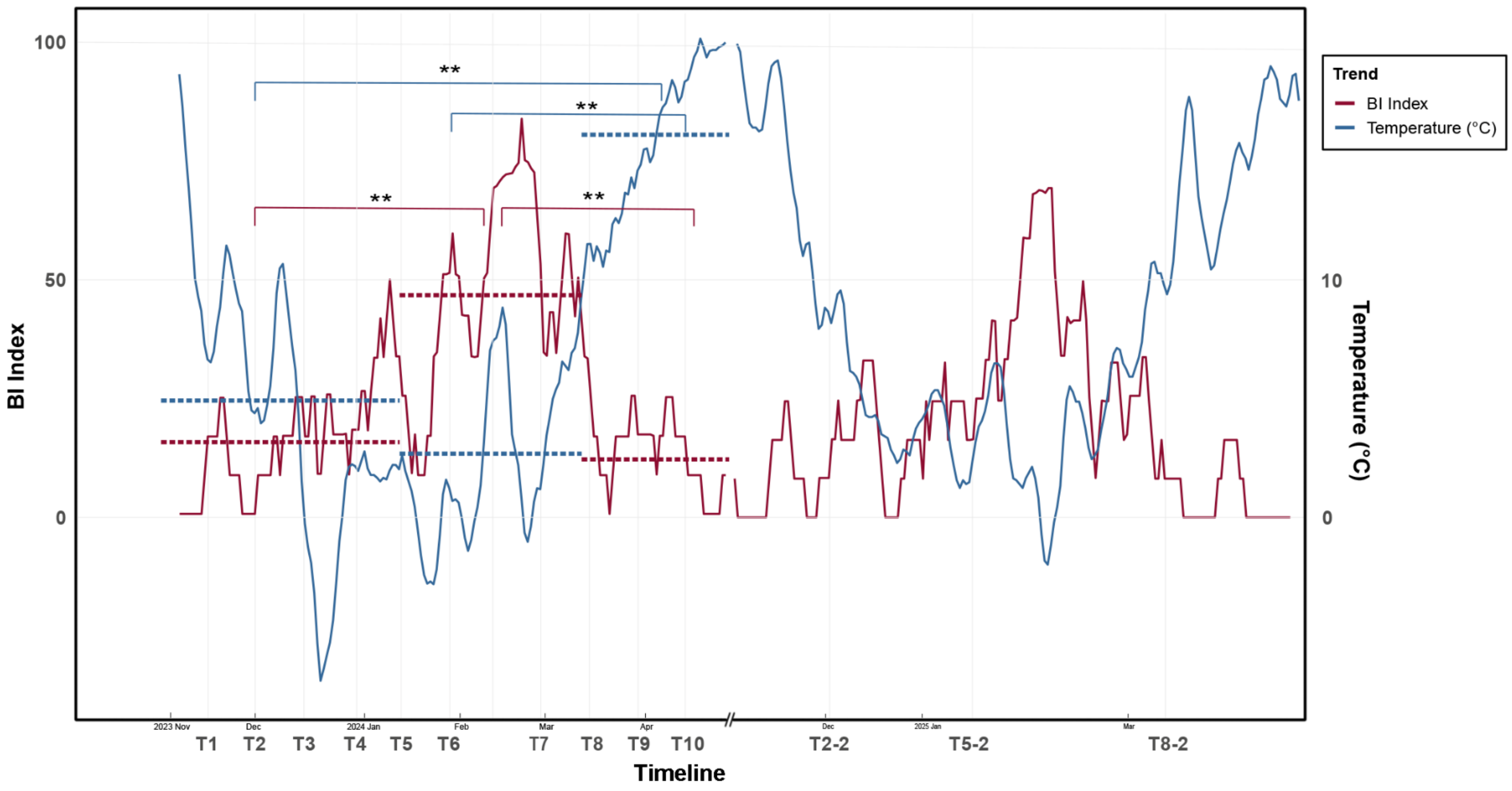

Figure S1. Dynamics of tourist intensity and ambient temperature during the overwintering period. Tourist intensity was quantified using 7-day moving averages of daily Baidu Index search volumes for the Chinese phrase "feeding seagulls" in Qingdao. Dashed horizontal lines indicate stage-specific mean values for each overwintering stage. Statistical significance was tested by Wilcoxon rank-sum test. *, $P < 0.05$.

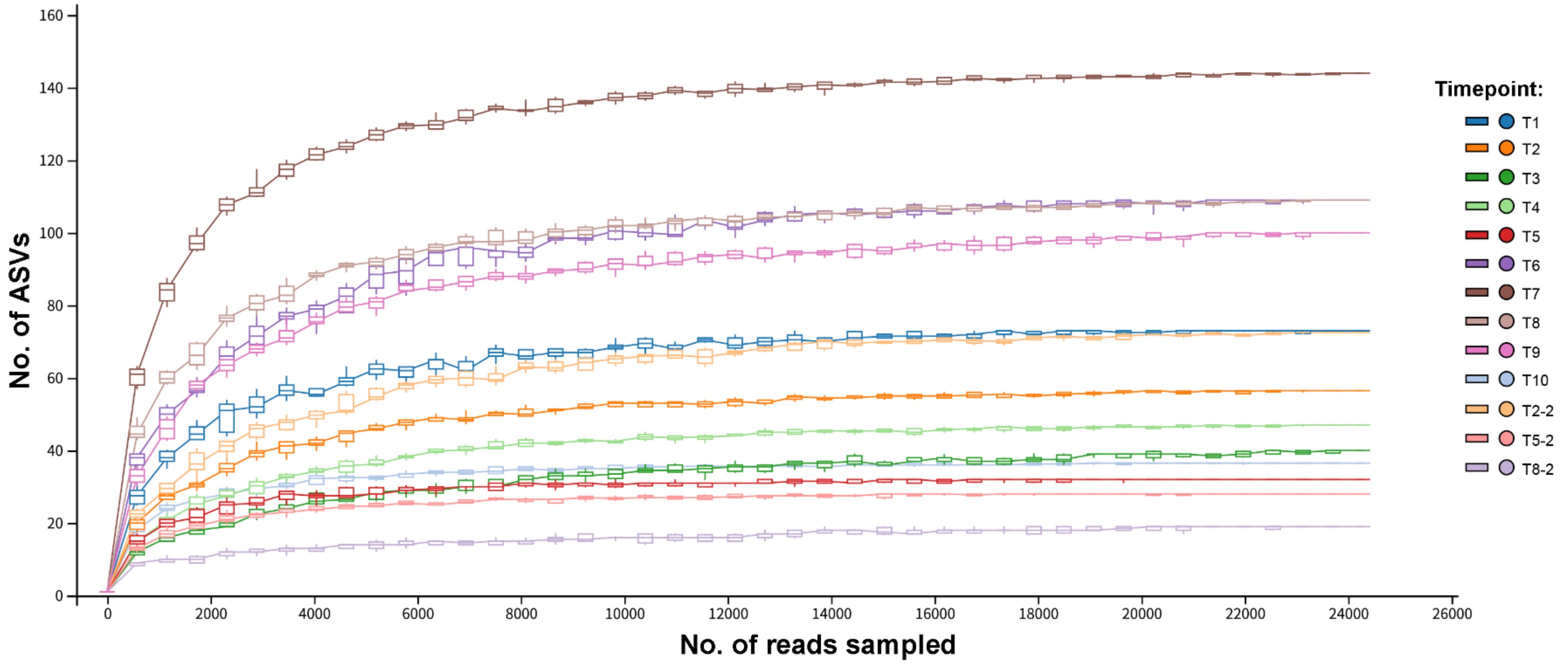

Figure S2. Rarefaction curves. The horizontal axis is the number of randomly selected sequences, and the vertical axis is the number of ASVs obtained based on the number of sequencing entries.

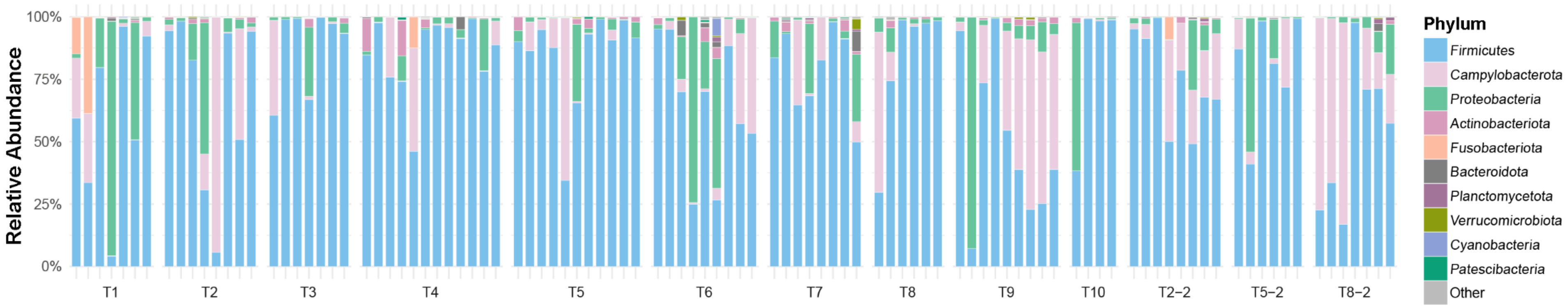

Figure S3. Relative abundance of the ten most abundant phylum.

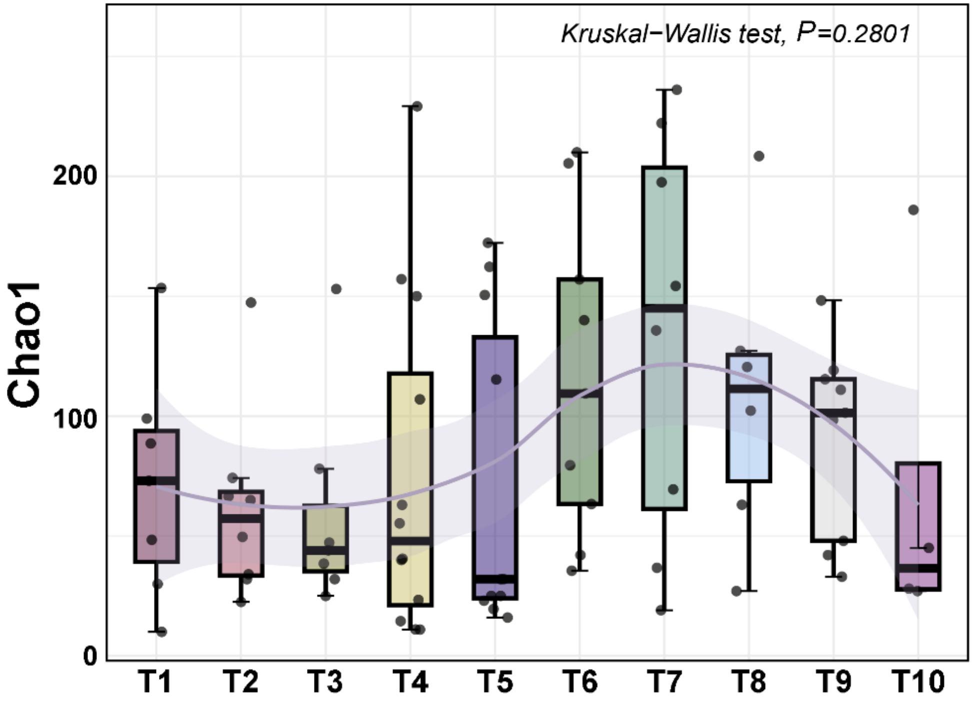

Figure S4. Alpha-diversity (Chao1) over time. Statistical significance was tested by Kruskal-Wallis test. The light purple trend line represents loess smooth regression estimates, with shaded cloud representing 95% confidence interval.

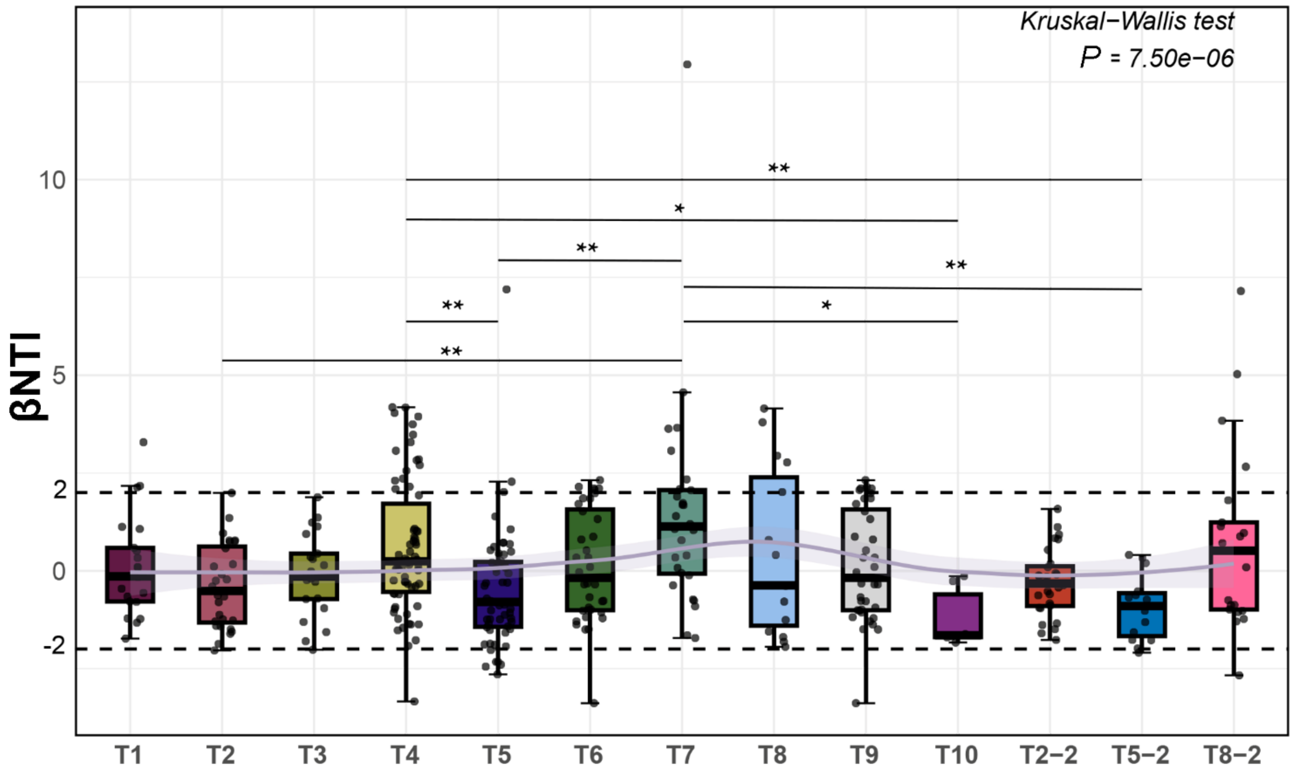

Figure S5. Box-plot of the total βNTI of sampling groups. Statistical significance was tested by Kruskal-Wallis test, followed by multiple comparisons using Wilcoxon rank-sum test (*, $P < 0.05$; **, $P < 0.01$).

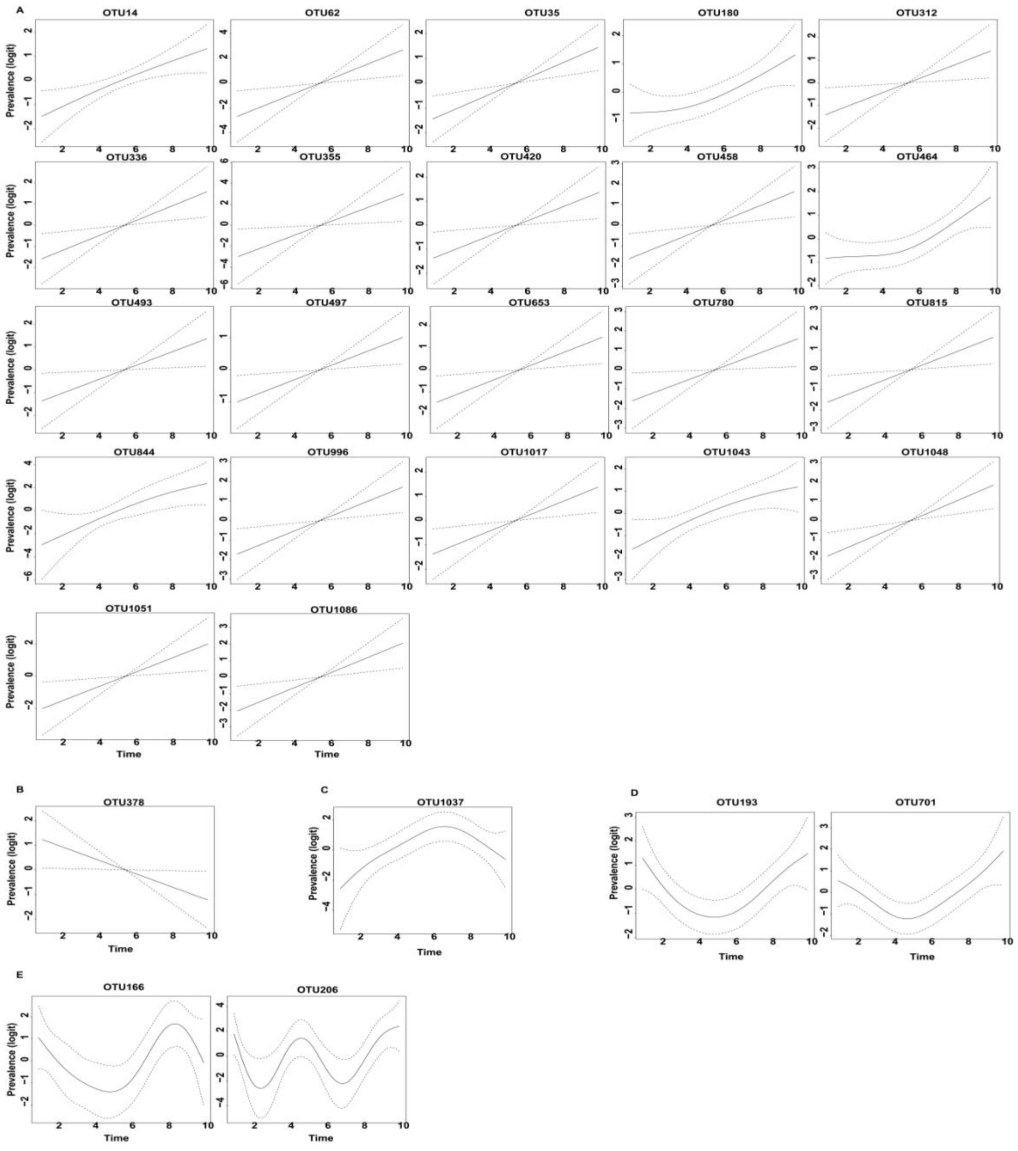

Figure S6. Temporal prevalence dynamics of representative species-level OTUs. Panels display five distinct patterns: (A) increasing, (B) decreasing, (C) hump-shaped, (D) U-shaped, and (E) oscillating.

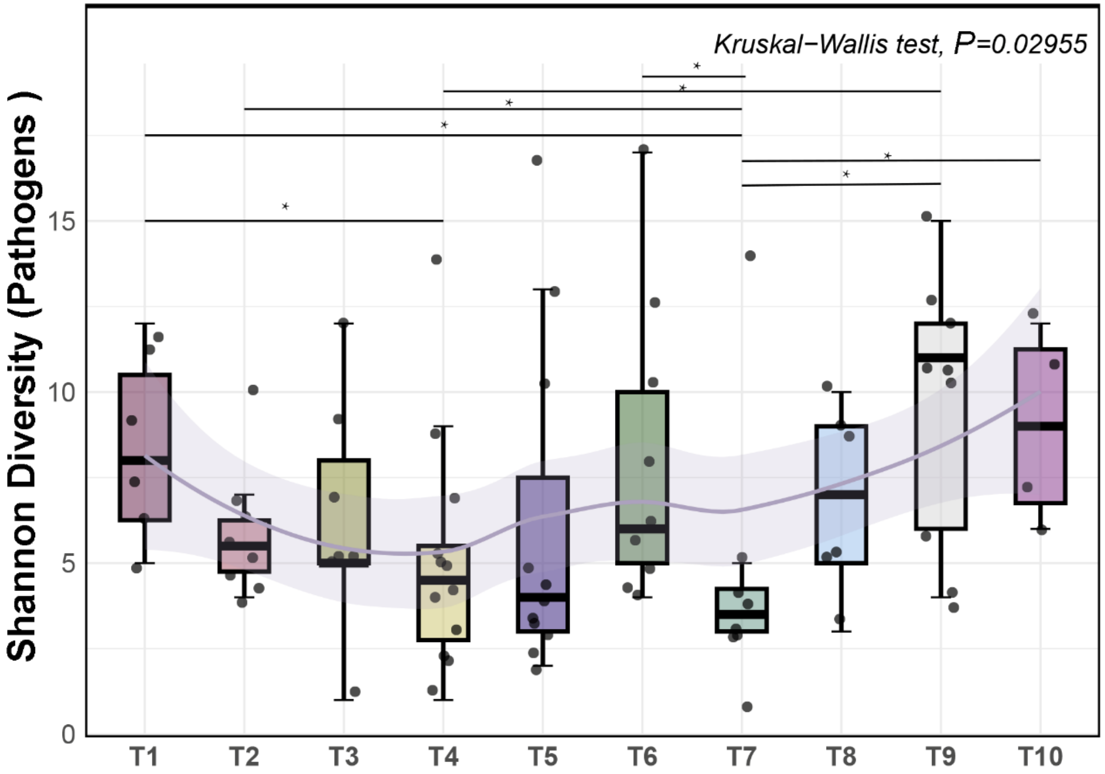

Figure S7. Alpha-diversity (Shannon) of putative pathogens over time. Statistical significance was tested by Kruskal-Wallis test, followed by multiple comparisons using Wilcoxon rank-sum test (*, $P < 0.05$). The light purple trend line represents loess smooth regression estimates, with shaded cloud representing 95% confidence interval.

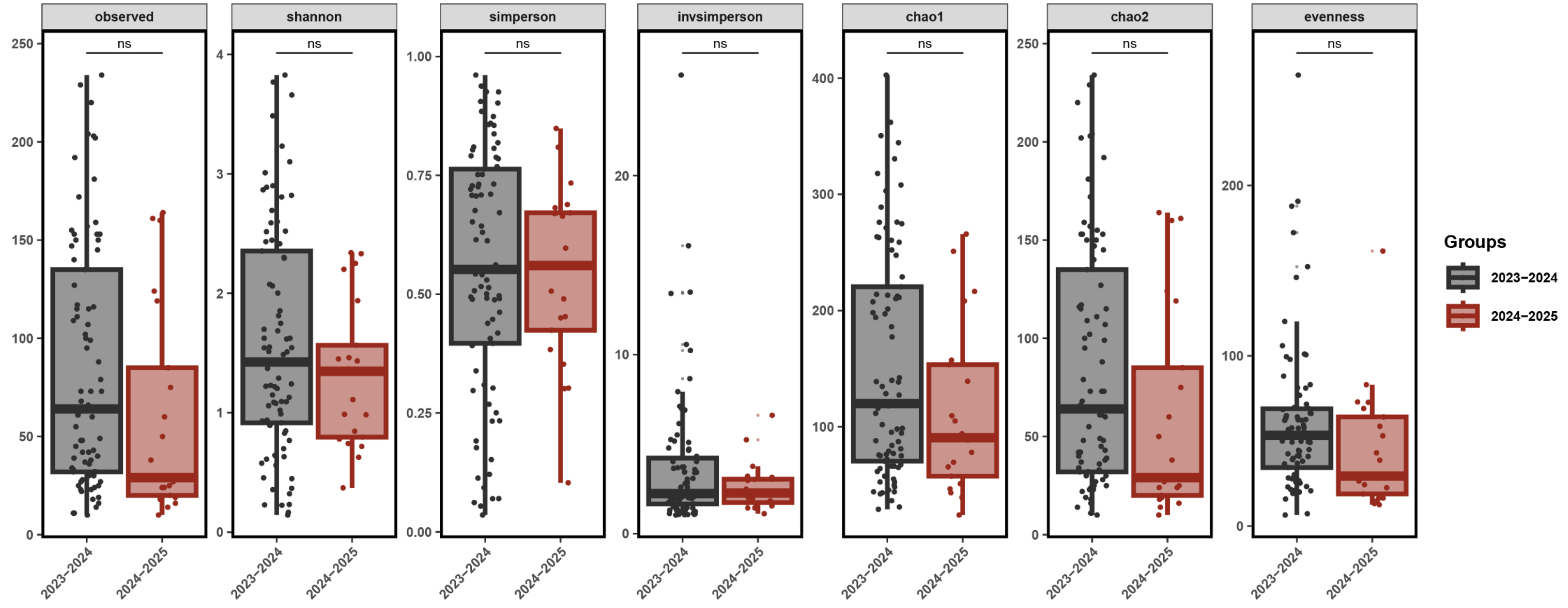

Figure S8. Alpha diversity analysis. Statistical significance was tested by Wilcoxon rank-sum test. NS, no significance.

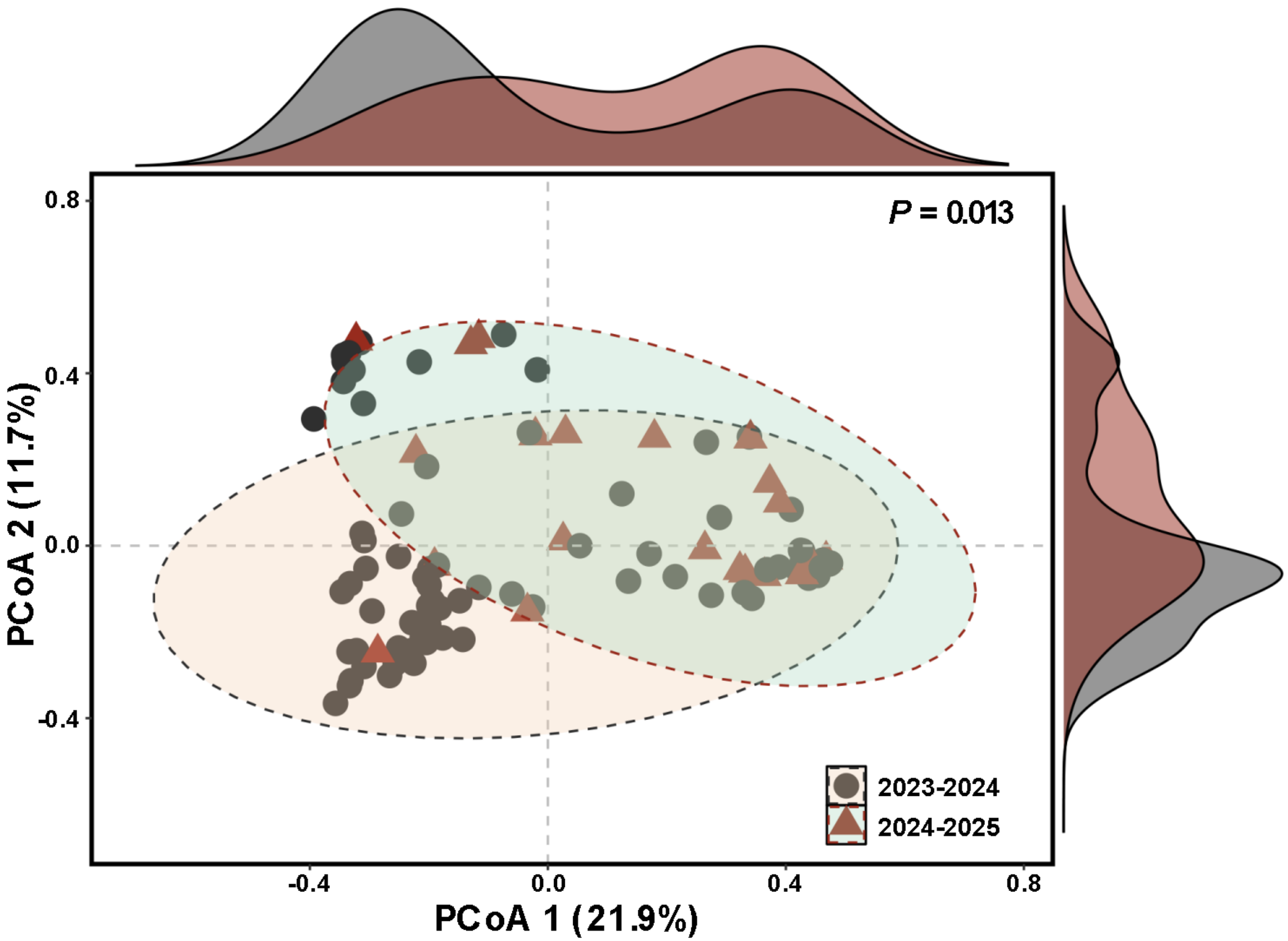

Figure S9. Interannual variation of the seagull fecal microbiome. The PCoA plot showing the Bray-Curtis distances of bacterial communities between initial (2023-2024) and subsequent (2024-2025) overwintering periods. *P*-value was obtained from PERMANOVA. The kernel density curves represent the distribution and peaks of sample densities along each axis.

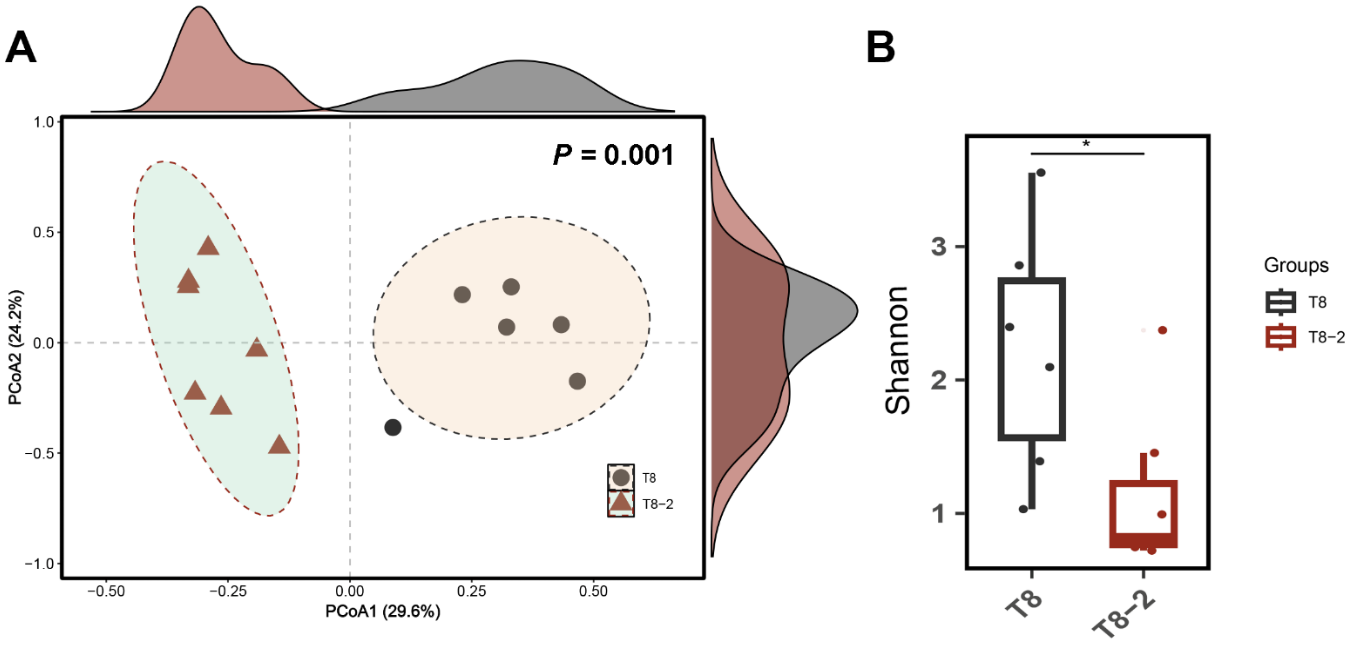

Figure S10. Significant divergences in both α- and β-diversity emerged between T8 and T8-2. (A) The PCoA plot based on Bray-Curtis dissimilarity distance showing the compositional differences. *P*-value was obtained from PERMANOVA. (B) Alpha-diversity (Shannon). Statistical significance was tested by Wilcoxon rank-sum test (*, $P < 0.05$)
